# Dietary ellagic acid therapy for CNS autoimmunity: Targeting on *Alloprevotella rava* and propionate metabolism

**DOI:** 10.1186/s40168-024-01819-8

**Published:** 2024-06-24

**Authors:** Bing Han, Lin Shi, Ming-Yue Bao, Feng-Lin Yu, Yan Zhang, Xin-Yu Lu, Yang Wang, Dong-Xiao Li, Jing-Chao Lin, Wei Jia, Xing Li, Yuan Zhang

**Affiliations:** 1 Key Laboratory of Medicinal Resources and Natural Pharmaceutical Chemistry (Shaanxi Normal University), The Ministry of Education; College of Life Sciences, Shaanxi Normal University, Xi’an, China; 2 College of Food Engineering and Nutritional Science, Shaanxi Normal University, Xi’an, China; 3 School of Medical Technology, Xi’an Medical University, Xi’an, 710021 Shaanxi China; 4 Metabo-Profile Biotechnology (Shanghai) Co. Ltd, Shanghai, 201315 China; 5 School of Chinese Medicine, Hong Kong Baptist University, Kowloon Tong, Hong Kong China

**Keywords:** Multiple sclerosis, Experimental autoimmune encephalomyelitis, Ellagic acid, Gut microbiota, *Alloprevotella rava*, Propionate

## Abstract

**Background:**

Mediterranean diet rich in polyphenolic compounds holds great promise to prevent and alleviate multiple sclerosis (MS), a central nervous system autoimmune disease associated with gut microbiome dysbiosis. Health-promoting effects of natural polyphenols with low bioavailability could be attributed to gut microbiota reconstruction. However, its underlying mechanism of action remains elusive, resulting in rare therapies have proposed for polyphenol-targeted modulation of gut microbiota for the treatment of MS.

**Results:**

We found that oral ellagic acid (EA), a natural polyphenol rich in the Mediterranean diet, effectively halted the progression of experimental autoimmune encephalomyelitis (EAE), the animal model of MS, via regulating a microbiota-metabolites-immunity axis. EA remodeled the gut microbiome composition and particularly increased the relative abundances of short-chain fatty acids -producing bacteria like *Alloprevotella*. Propionate (C3) was most significantly up-regulated by EA, and integrative modeling revealed a strong negative correlation between *Alloprevotella* or C3 and the pathological symptoms of EAE. Gut microbiota depletion negated the alleviating effects of EA on EAE, whereas oral administration of *Alloprevotella rava* mimicked the beneficial effects of EA on EAE. Moreover, EA directly promoted *Alloprevotella rava* (DSM 22548) growth and C3 production in vitro. The cell-free supernatants of *Alloprevotella rava* co-culture with EA suppressed Th17 differentiation by modulating acetylation in cell models. C3 can alleviate EAE development, and the mechanism may be through inhibiting HDAC activity and up-regulating acetylation thereby reducing inflammatory cytokines secreted by pathogenic Th17 cells.

**Conclusions:**

Our study identifies EA as a novel and potentially effective prebiotic for improving MS and other autoimmune diseases via the microbiota-metabolites-immunity axis.

Video Abstract

**Supplementary Information:**

The online version contains supplementary material available at 10.1186/s40168-024-01819-8.

## Background

Multiple sclerosis (MS) is an autoimmune inflammatory demyelination disease. Although the pathogenesis has not been fully elucidated, the development of MS is associated with environmental, genetic, and immune dysregulation [1–3]. T helper 17 (Th17) cells could contribute to central nervous system (CNS) inflammation development in MS patients by recruiting other immune cells through the production of inflammatory factors [4].

Gut microbiota plays an essential role in the pathogenesis of MS [5, 6]. Transplantation of feces from MS patients into germ-free mice increased the likelihood of inducing experimental autoimmune encephalomyelitis (EAE), a validated animal model of MS [7], underscoring the importance of gut microbiota interactions in autoimmune diseases. This study critically implies that modulation of the gut microbiota may influence autoreactive T cells, thereby affecting MS pathogenesis. Until now, there are however no therapeutic drugs specifically targeting the regulation of gut microbiota for the treatment of autoimmune diseases.

*Alloprevotella* are mainly enriched in the human oral cavity, but many "species" remain undescribed and poorly understood [8]. *Alloprevotella rava* an anaerobic Gram-negative bacillus isolated from the human oral cavity [9], has been reported to be enriched in salivary microorganisms of periodontitis patients while was lower in patients with Behçet' s disease than in healthy controls [10, 11]. *Alloprevotella rava* exhibited a glycolytic effect and ferments to produce acetic acid and succinic acid, a crucial compound involved in propionate production [9, 12]. There are no relevant reports indicating the role of *Alloprevotella rava* in treatment of MS and other autoimmune diseases.

The Mediterranean diet (MedDiet) primarily consists of vegetables, fruits, fish, grains, beans, and olive oil and has been associated with lower risk of metabolic diseases[13, 14]. A recent intervention study conducted in southern Italy has demonstrated the gut microbiota-mediated beneficial effects of the Mediterranean diet (MedDiet) on patients with MS [15]. Moreover, MedDiet has shown to greatly increased the concentration short chain fatty acids (SCFAs) [16], the diversity of gut microbiota and balanced the ratio of Th17 cells to Treg [17]. Notably, ellagic acid (EA), a natural plant polyphenol abundantly presented in fruits, vegetables, and nuts that are part of the MedDiet. Oral EA has very low bioavailability, and needs to be converted into more easily absorbed urolithin metabolites by gut microbiota. Although urolithin A (URA) is the major active microbial metabolite of EA in the intestine and could alleviate the disease course of EAE [18, 19], our study demonstrated that EA treatment resulted in a more pronounced improvement compared to URA when the same dose of EA or URA was administered to EAE mice. These data suggested EA may exert therapeutic effects on EAE independently of URA, which has not been clarified.

In the present study, we conducted a thorough investigation to assess the influence and underlying mechanisms of oral EA on a representative rodent model of multiple sclerosis (EAE). Our findings demonstrate that oral EA effectively mitigated the disease course of EAE via increasing the relative abundance of SCFAs-producing bacteria i.e., *Alloprevotella*, *Bacteroides*, and *Prevotella*, and fecal contents of SCFAs, particularly propionate (C3). Integrative multi-omics analyses demonstrated a strong negative correlation between *Alloprevotella*, C3 and improved pathological symptoms of EAE. More specifically, EA stimulated the growth *Alloprevotella rava* and directly enhanced the production of C3. The cell-free supernatants from the co-culture of *Alloprevotella rava* with EA successfully inhibited Th17 differentiation by up-regulating acetylation modification and the recolonization of EAE mice with *Alloprevotella rava* also resulted in significant improvement of EAE symptoms. Consistently, oral administration of C3 mimicked the activity of EA, which suppressed Th17 cell differentiation by inhibiting histone deacetylase (HDAC) and up-regulating acetylation modification, ultimately attenuating EAE disease progression. Collectively, our study innovatively identifies novel mechanisms underlying the beneficial effects of EA on combating MS and other autoimmune diseases via the microbiota-metabolites-immunity axis.

## Methods

A complete description of the methods and associated references are provided in Materials and Methods in the Supplementary Material. The EAE model was treated with EA and C3. All experimental procedures and protocols using mice were approved by the Committee on the Ethics of Animal Experiments of Shaanxi Normal University (No. SCXK-2018–001) and were carried out in accordance with the approved institutional guidelines and regulations. C57BL/6 mice (6–8 weeks of age) were purchased from the Xi'an Jiaotong University (Xi’an, China). The reagents and experimental procedure for histopathological examination, preparation of infiltrating MNCs in CNS, fecal microbiota transplantation and antibiotic treatment, CD4^+^ T cells isolation and culture, cytokine measurement by ELISA, FACS analysis, HDAC activity assay, western blotting analysis, 16S rRNA microbial sequencing analysis, fecal sample preparation and targeted metabolisms analysis, integrative analysis, and other experiments mentioned in this paper are described in the Materials and Methods in the supplemental material.

## Results

### Oral administration of EA alleviated EAE

**Fig. 1:**
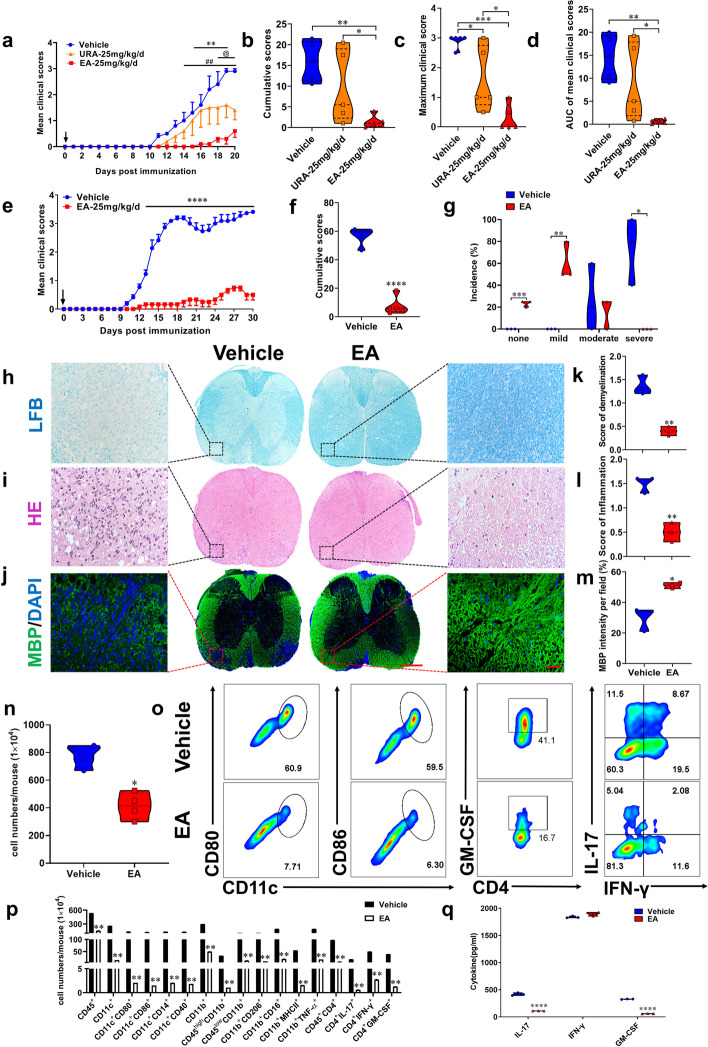
Oral EA treatment significantly alleviated EAE. C57BL/6 mice were immunized with MOG_35-55_, and fed with the same dose of vehicle, EA or URA (25 mg/kg) by oral gavage daily, starting from day 0 p.i. as indicated by the arrow. **a** Disease was scored daily on a 0–5 scale. ## indicates the significant difference between Vehicle and EA from day 14 p.i. to 20 p.i. (*p* < 0.01). @ indicates the significant difference between Vehicle and URA from day 18 p.i. to 20 p.i. (*p* < 0.05). ** indicates the significant difference between EA and URA from day 15 p.i. to 19 p.i. (*p* < 0.01). **b** Cumulative scores of EAE (sum of daily clinical scores). **c** Maximum clinical score. **d** AUC of clinical scores. Data are expressed as mean ± SEM (*n* = 5 mice in each group). **e** Disease severity was scored daily on a 0–5 scale. Arrow indicates the time point of treatment. **f** Cumulative scores were the total daily clinical scores from (e). **g** Incidence of disease severity at the peak of the experiment when treatment. Disease severity is graded as severe (clinical score: > 3), moderate (clinical score: 1.5–3), mild (clinical score: < 1.5), or none (no clinical signs). **h**-**j** EA- or vehicle-treated EAE mice described in (e) were sacrificed at day 30 p.i., and spinal cords were harvested. Spinal cords sections were analyzed by Luxol fast blue (LFB, for degree of demyelination), haematoxylin and eosin (H&E, for inflammation), and MBP immunofluorescence staining. Full slice bar = 500 μm, partial slice bar = 50 μm. **k**-**m** Statistical analysis of (**h**-**j**). (n) Total numbers of mononuclear cells (MNCs) in the CNS. **o**-**p** The percentages and absolute numbers of various MNC subpopulations were measured by flow cytometry. **q** Supernatants derived from the splenocytes of mice described in (e) were cultured with MOG_35-55_ were analyzed for the levels of indicated cytokines. Data are expressed as mean ± SEM (*n* = 5 mice in each group), *p < 0.05, **p < 0.01, ***p < 0.001 and ****p < 0.0001, determined by two-way ANOVA (a-d, e), unpaired Student’s *t*-test (f, k-n, p), or Multiple t’ tests (g, q). One representative of three independent experiments is shown

EA, with its low bioavailability, is extensively metabolized by gut microbiota into urolithins, which are found in high concentrations in plasma and urine, conferring numerous health benefits [18, 19]. To clarify preventive effects of oral EA and its major endogenous metabolite URA against EAE, we first compared their protective effects under the same conditions. The results shown that oral EA improves clinical signs of EAE mice more significantly than its derivantive URA at the same dose (Fig. 1a-d), indicating its unique function independent of URA.

EAE mice was then intragastrically administered with different doses of EA (e.g., 5, 10, or 25 mg/kg/day) by oral gavage starting at day 0 post-immunization (p.i.). The ameliorative effects of EA on EAE were dose-dependent, with 25 mg/kg/day completely suppressing EAE (Figure S1a-h). We thus used this dose for subsequent studies. EA reduced EAE clinical scores and disease severity compared with the vehicle-treated group (Fig. 1e-f). At the peak of the disease, the majority of EA-treated EAE mice displayed none or mild clinical signs, while the severe disease was mostly observed in the vehicle-treated mice (Fig. 1g). To assess effects of EA on EAE-associated CNS pathology, spinal cords were obtained from EA- and vehicle-treated mice for histological analysis. As shown in Fig. 1h-m, EA significantly reduced inflammation and demyelination of EAE mice. Also, a remarkable loss of myelin basic protein (MBP) was observed in the vehicle-treated group, whereas MBP was not injured in EA-treated EAE mice (Fig. 1h-m). These data suggest that oral EA reduced myelin damage while enhancing remyelination of EAE mice.

We investigated the number and composition of infiltrating mononuclear cells (MNCs) in the CNS. The total number of MNCs was reduced in the CNS of EA-treated mice compared with vehicle-treated mice (Fig. 1n). EA significantly reduced percentage and an absolute number of CD45^+^ leukocytes, co-stimulatory molecules (e.g., MHC class II, CD80, and CD86) and activated markers (e.g., CD14^+^, CD206, CD40, and TNF-α) of antigen-presenting cells (CD11b^+^ and CD11c^+^), as well as decreased IFN-γ^+^, IL-17^+^, and GM-CSF^+^ CD4^+^ T cells (Fig. 1o-p, Figure S2a-j). Consistently, MOG-induced inflammatory cytokines, such as IL-17 and GM-CSF, were significantly reduced in the splenic culture of EAE mice (Fig. 1q). These results indicate that EA suppressed CNS inflammation and the secretion of pro-inflammatory cytokines.

To further elucidate the underlying mechanisms driving EA to ameliorate EAE symptoms, we performed RNA-sequence of spinal cord from EA- and vehicle-treated EAE mice. Principal component analysis (PCA) revealed distinct sample dispersion between vehicle and EA groups (Figure S3a). A total of 4260 genes differentially expressed between EA- and vehicle-treated EAE mice, including 1536 up-regulated genes and 2724 down-regulated genes (Figure S3b, c). Among them, Th17 differentiation-related genes, such as IL-6, TGF-β and IL-17f, were down-regulated in the EA-treated group (Figure S3d). GO and KEGG enrichment analysis suggests that immune system and Th17 differentiation signaling mediate the ameliorative effect of EA on EAE (Figure S3e, f).

### EA modulated composition of gut microbes in EAE mice

**Fig. 2:**
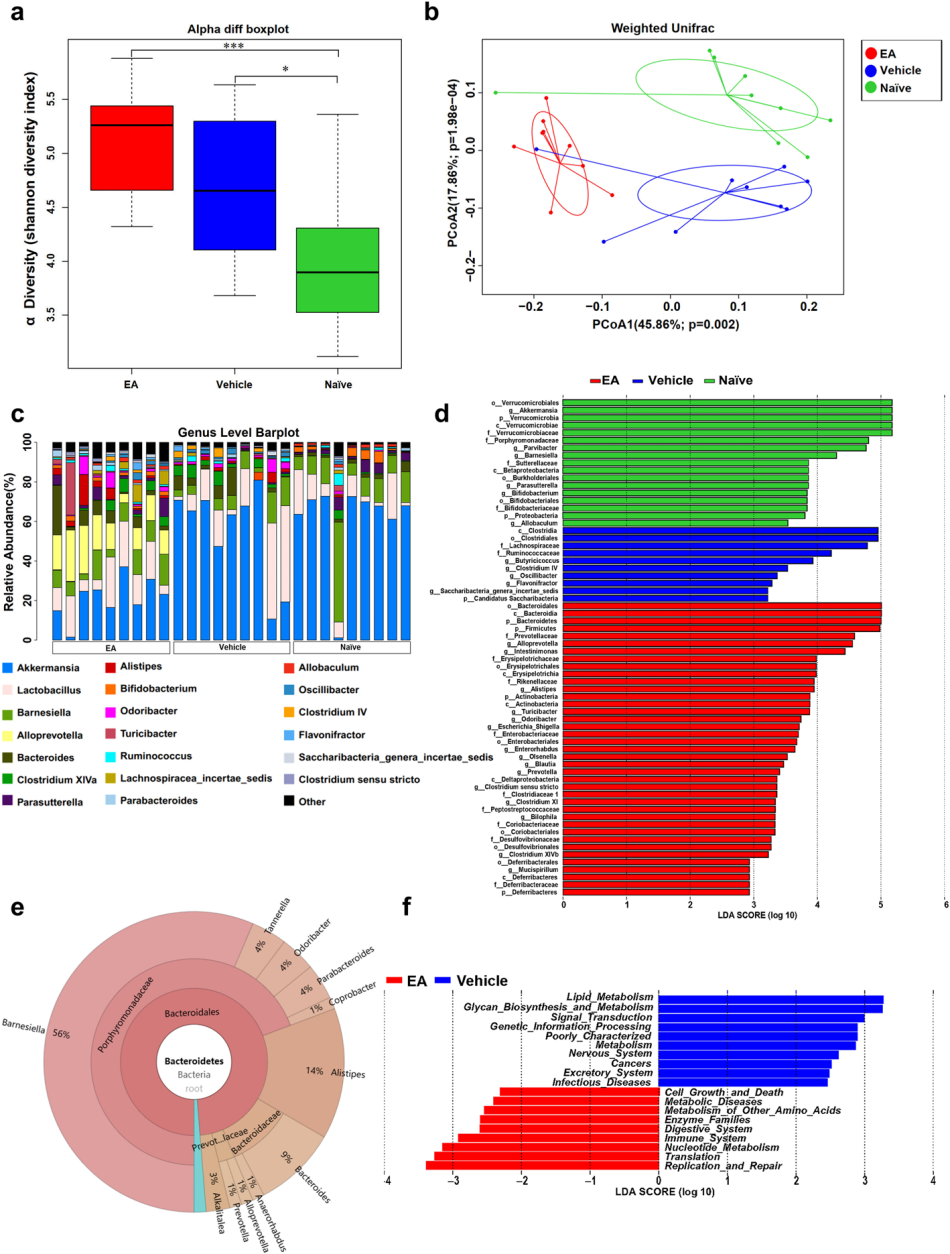
EA treatment altered the gut microbial composition in EAE mice. Stool samples from naïve, vehicle- and EA-treated groups collected at day 17 ~ 19 days p.i. were subjected to 16S rRNA sequencing (*n* = 9 in each group). **a** Shannon diversity index of naïve, vehicle and EA subjects. **b** PCoA analysis of naïve, vehicle and EA subjects (Adonis test: *p* = 0.001 *R*^2^ = 0.387). **c** Bar plot of composition and relative abundance of genus level in the gut microbiota of three groups. **d** LDA was analyzed and the LDA score > 2 was displayed. **e** Species annotation knots KRONA schematic. **f** LEfSe analysis statistics KEGG differential pathways

To analyze the overall structural changes of gut microbiota influenced by EA, stool samples collected from the different groups and taxa were determined using 16S rRNA sequencing. Compared with naïve control, the total number of operational taxonomic units (OTUs) increased in both vehicle- and EA-treated EAE mice (Figure S4a). A total of 379 OTUs were identified in the three groups, with 2, 18, and 10 OTUs were unique to the naïve, vehicle and EA groups, respectively (Figure S4b). The alpha diversity of gut microbiota was increased in both the vehicle and EA group relative to the naïve control (Fig. 2a). The principal co-ordinates analysis (PCoA) results indicated the major differences in the compositions of gut microbiota among groups (Fig. 2b). EA remodeled the gut microbiota composition of EAE mice. For instance, at the phylum level, the percentage of the Bacteroidetes in the vehicle group was down-regulated compared with the naïve group, while was up-regulated by EA. EA increased Firmicutes while decreasing Verrucomicrobia compared with the vehicle and the naïve groups (Figure S4c). EA increased the accumulation of genus *Alloprevotella*, phylum Firmicutes, family Prevotellaceae, and several taxa belonging to Bacteroidetes, while diminishing the relative abundance of class Clostridia, order Clostridiales, order Verrucomicrobiales, genus *Akkermansia*, class Verrucomicrobia and family Verrucomicrobiaceae (Fig. 2c-d). The genus *Alloprevotella* belongs to Bacteroidetes, accounting for a relative proportion of approximately 1% (Fig. 2e). KEGG results showed that metabolic pathways significantly differentially affected by EA treatment were enriched in metabolic diseases and immune system pathways (Fig. 2f).

### Oral EA altered the fecal metabolome in EAE mice

**Fig. 3:**
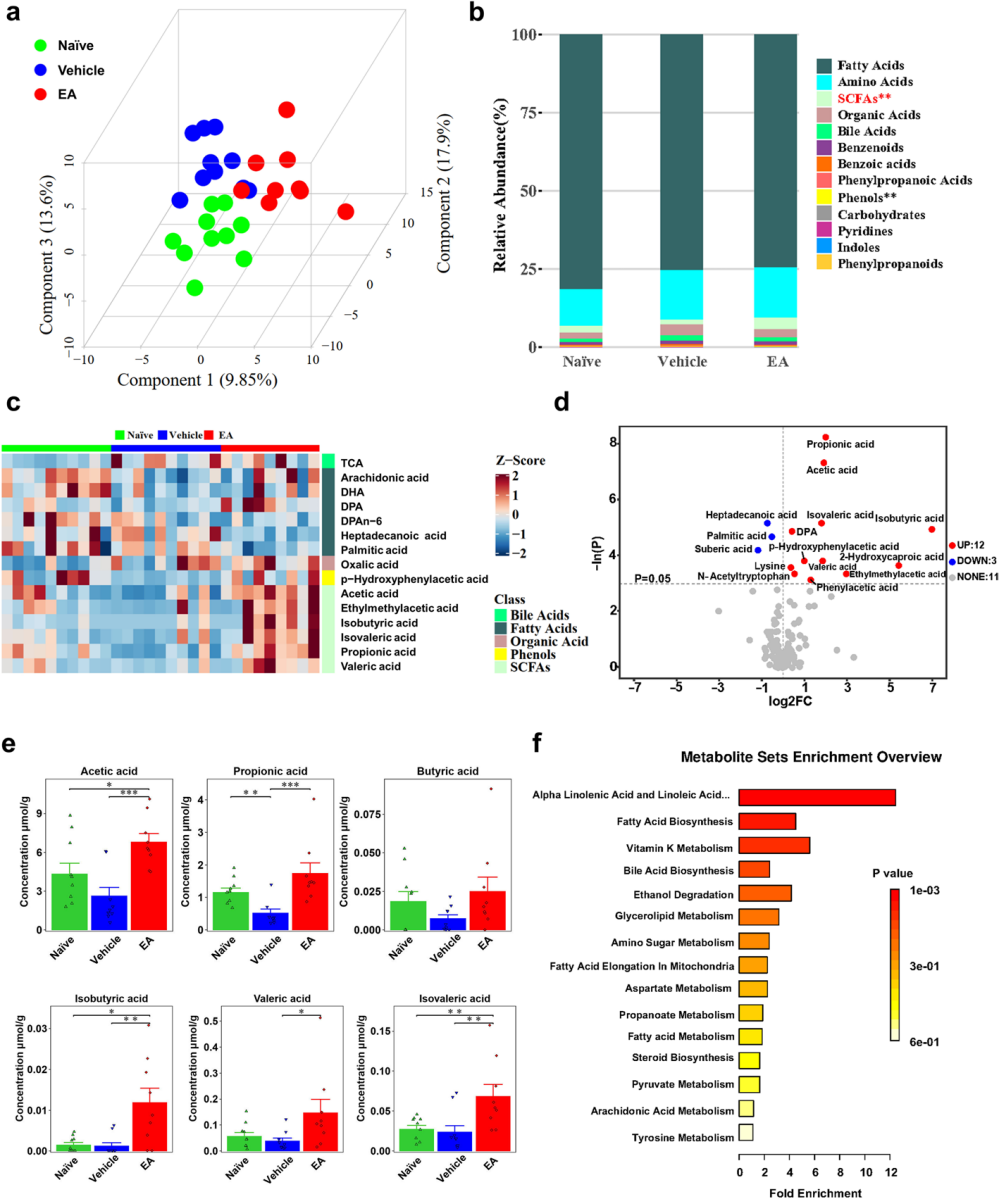
EA treatment altered the metabolite composition of gut microbiota in EAE mice. Stool samples from naïve, vehicle- and EA-treated groups collected at day 17 ~ 19 days p.i. were subjected to metabolically analyzed (*n* = 9 in each group). **a** PLS-DA analysis among three groups. **b** The relative abundance of various metabolites between the three groups. **c** Heatmap of the scaled abundance of 15 differential metabolites. **d** Volcano Plot of the significance of metabolic changes between two samples. **e** Analysis of significant differences between three groups of short chain fatty acids. **f** Enriched metabolic pathways involved with the significantly different metabolites

Given the significant influence of oral EA on gut microbiota composition and the fecal metabolism serving as a functional indicator of microbial activity, we next conducted a targeted metabolomics analysis of fecal samples to investigate changes in gut microbiota-related metabolites among EA-treated EAE mice. Fecal metabolome differed between three groups (Fig. 3a). A total of 132 metabolites, including fatty acids, SCFAs, bile acids, indoles, and amino acids, were detected (Fig. 3b). EA statistically increased 12 metabolites while decreasing 3 metabolites compared with the vehicle (Fig. 3c). Compared with the naïve group, acetic acid, propanoic acid, butyric acid, isobutyric acid, valeric acid, and isovaleric acid were decreased in the vehicle-treated EAE mice. EA significantly up-regulated SCFAs relative to vehicle control (Fig. 3d). Propionate (C3) was predominantly elevated in EA-treated mice (Fig. 3e). Differential metabolites were enriched in pathways related with fatty acid biosynthesis and propanoate metabolism (Fig. 3f).

### Integrated modeling of gut microbiota composition, fecal metabolite levels, and pathological markers in EAE associated with EA treatment

**Fig. 4:**
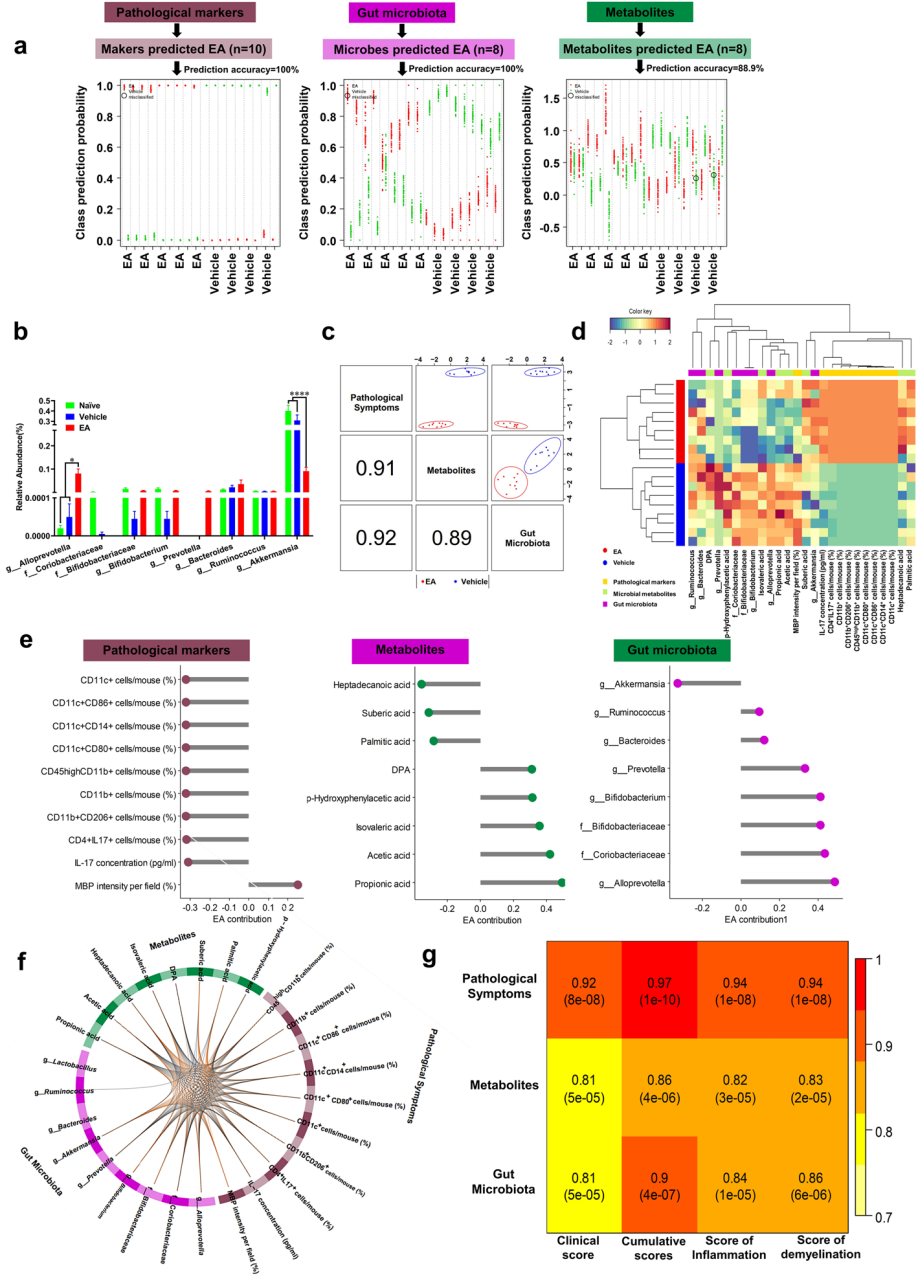
Integrative modeling on gut microbiota compositions, fecal levels of metabolites and pathological markers of EAE in relation to EA treatment. **a** The performance of predictive models for three optimally identified parsimonious sets of predictors on EA treatment. Predictors include: 10 markers reflecting pathological symptoms of EAE, 8 microbial species and 8 microbial metabolites in fecal samples that were altered by EA. The random forest algorithm incorporated into a repeated double cross validation framework with unbiased variable selection was applied to effectively determine a parsimonious set of features from pathological symptoms markers of EAE mice (*n* = 24), gut microbiota (*n* = 65), and microbial metabolites (*n* = 132) that could predict EA from EAE. In the figure, each swim lane represents one mice sample. For each sample, class probabilities were computed from 100 double cross-validations. Class probabilities are color coded by class and presented per repetition (smaller dots) and averaged over all repetitions (larger dots). Misclassified samples are circled. Predictive accuracy was calculated as a number of correctly predicted samples/total number of measured samples. **b** Analysis of key predicted gut bacterial relative abundance differences between the three groups of mice (*n* = 9 in each group). **c** The model performance of DIABLO integrative modeling on the three types of predictors in relation to EA treatment. The use of full weight design matrix in DIABLO maximized the correlated information between multiple data sets. The scatter plot displays the clustering of groups, i.e., EA and EAE (upper diagonal plot) and correlations between components (lower diagonal plot). **d** The Circos plot shows the positive (negative) correlation, denoted as brown (gray) lines, between selected predictors. **e** A clustered image map (Euclidean distance, complete linkage) of the selected predictors. Samples are represented in rows, selected features on the first component in columns. **f** Contributions of selecting predictors from each dataset on predicting EA. Loading of DIABLO integrative modeling for each predictor is presented. **g** Heatmap shows correlations between integrated key features and clinical disease scores reflecting disease progression, CNS inflammatory cell infiltration and demyelination these disease pathological features. Data are expressed as mean ± SEM, *p < 0.05 and ****p < 0.0001, determined by two-way ANOVA

We assessed the interplay across gut microbiota compositions, fecal levels of metabolites, and pathological markers of EAE, to demonstrate potential connection. A total of 10, 8, and 8 markers of pathological symptoms of EAE, gut microbiota compositions, and fecal levels of microbial metabolites were optimally identified by using predictive modeling. When assessing individually, a significant separation between EA- and vehicle-treated EAE mice was achieved with a predictive accuracy of 100%, 100%, and 89% for pathological markers of EAE, gut microbiota, and metabolites, respectively (Fig. 4a). The key gut microbiota predictors included genus *Alloprevotella*, family Coriobacteriaceae, family Bifidobacteriaceae, genus *Bifidobacterium*, genus *Prevotella*, genus *Bacteroides*, genus *Ruminococcus*, and genus *Akkermansia* (Fig. 4b)*.* The key microbial metabolite was propionic acid, which has been reported to exhibit multiple biological functions related to metabolic disturbances, inflammation, and cardiovascular diseases [20–22] (Supplementary Table 1). Integrating three panels of maximally correlated key features greatly discriminated the EA group of vehicle control (Fig. 4c-d). Of note, we found that *Alloprevotella* and C3 were the leading contributors to the discrimination of the EA group from the vehicle-treated group, and both of which were highly negatively correlated with pathological markers of EAE (Fig. 4e). Furthermore, having demonstrated the inter-relationship of gut microbiota compositions, metabolites, and pathological symptoms that were beneficially affected by EA (Fig. 4f), we demonstrated strong correlations between integrated key predictors and clinical disease scores that reflect disease progression, CNS inflammatory cell infiltration and demyelination (Fig. 4g). Collectively, these data suggest core roles for the gut microbiota, i.e. *Alloprevotella* and the microbial metabolite C3 in EA -mediated treatment of EAE.

### Gut microbiota was essential for EA to alleviate EAE

**Fig. 5:**
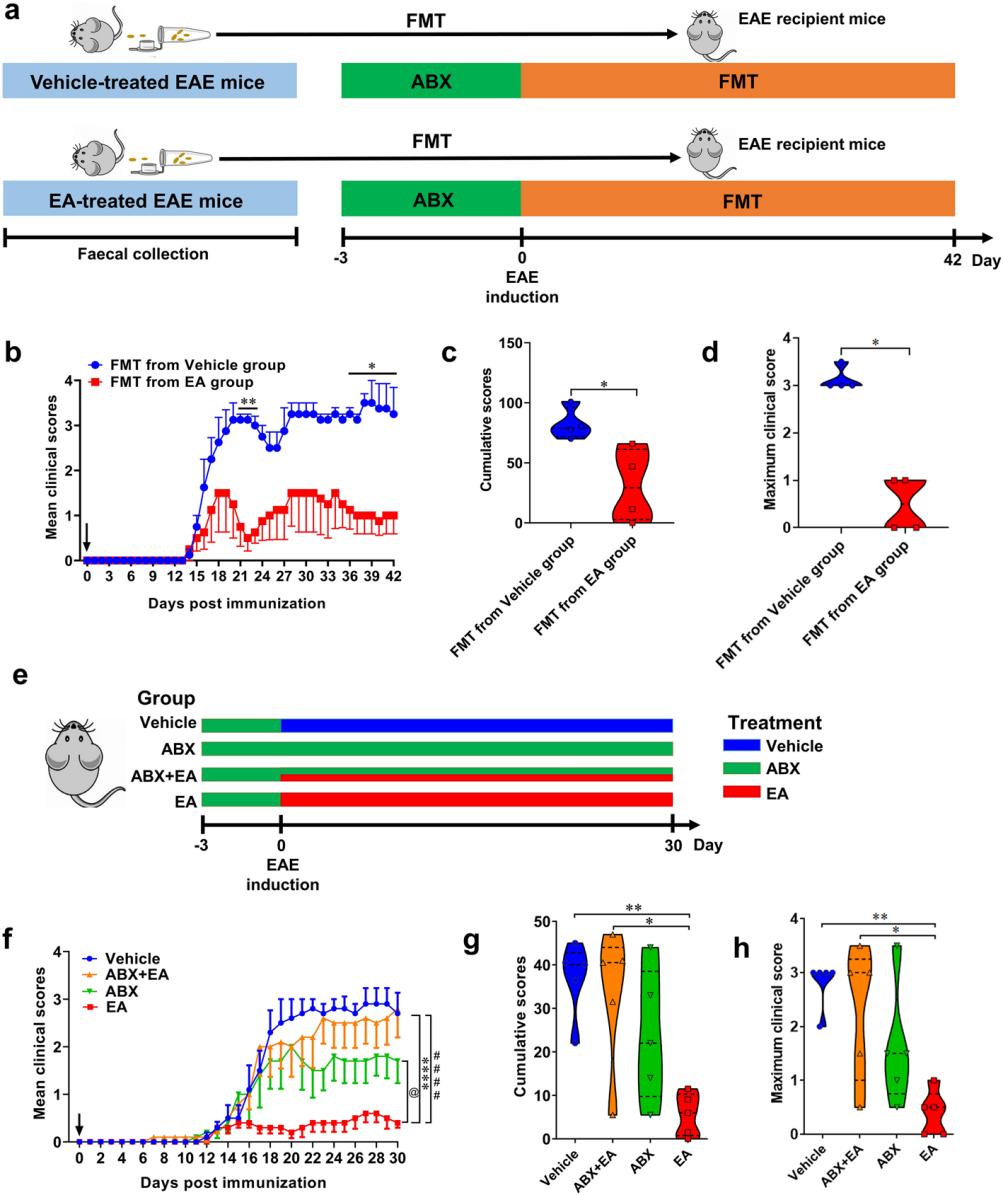
Therapeutic effects of EA were mediated by gut microbiota. **a** Schematic diagram of fecal bacteria transplantation experiment in PBS-treated and EA-treated groups. WT mice were put on a course of intragastric antibiotics administration for 3 days for gut microbiota depletion prior to Fecal microbiota transplantation (FMT) [55]. Feces of EAE mice treated with vehicle or EA were collected, and performed FMT on recipient mice. FMT was administered daily from the day of immunization. **b** Mean clinical scores. **c** Cumulative scores of EAE (sum of daily clinical scores). **d** Maximum clinical score. **e** Experimental schematic illustration of the effect of antibiotic depletion of gut microbiota on the effects of EA treatment in EAE. C57BL/6 mice were immunized with MOG_35-55_ and treated daily with vehicle, ABX + EA (25 mg/kg/day), ABX, or EA (25 mg/kg/day) by oral gavage. **f** Mean clinical scores. #### indicates the significant difference between vehicle and EA (*p* < 0.0001). **** indicates the significant difference between ABX + EA and EA (*p* < 0.0001). @ indicates the significant difference between ABX and EA (*p* < 0.05). **g** Cumulative scores of EAE (sum of clinical scores from day 17 to day 30). (h) Maximum clinical score. Data are expressed as mean ± SEM (*n* = 4–5 mice in each group), *p < 0.05, **p < 0.01 and ****p < 0.0001, determined by two-way ANOVA (b, f, g, h), or unpaired Student’s *t*-test (c, d). One representative of three independent experiments is shown

We performed fecal microbiota transplantation experiment to conform the vital role of gut microbiota in mediating the therapeutic effect on EAE. Fresh feces were collected from vehicle- and EA-treated EAE mice, and fecal supernatants were orally administered daily to recipient mice (Fig. 5a). Compared with mice that received fecal microbiota from the vehicle group, those that received from EA remarkably alleviated disease severity (Fig. 5b). The cumulative clinical score and peak clinical scores were reduced (Fig. 5c, d).

We also tested whether EA could alleviate EAE in a quadruple antibiotic (ABX) treated EAE mice (Fig. 5e). Gut microbiota depletion partly removed the alleviating effects of EA on clinical score (Fig. 5f) and cumulative score of EAE mice (Fig. 5g), as well as a significant decrease in the peak clinical scores (Fig. 5h). These data conformed that gut microbiota plays a crucial role in enabling EA to exert its therapeutic effect on EAE.

### Oral administration of *Alloprevotella rava* significantly alleviated EAE

**Fig. 6:**
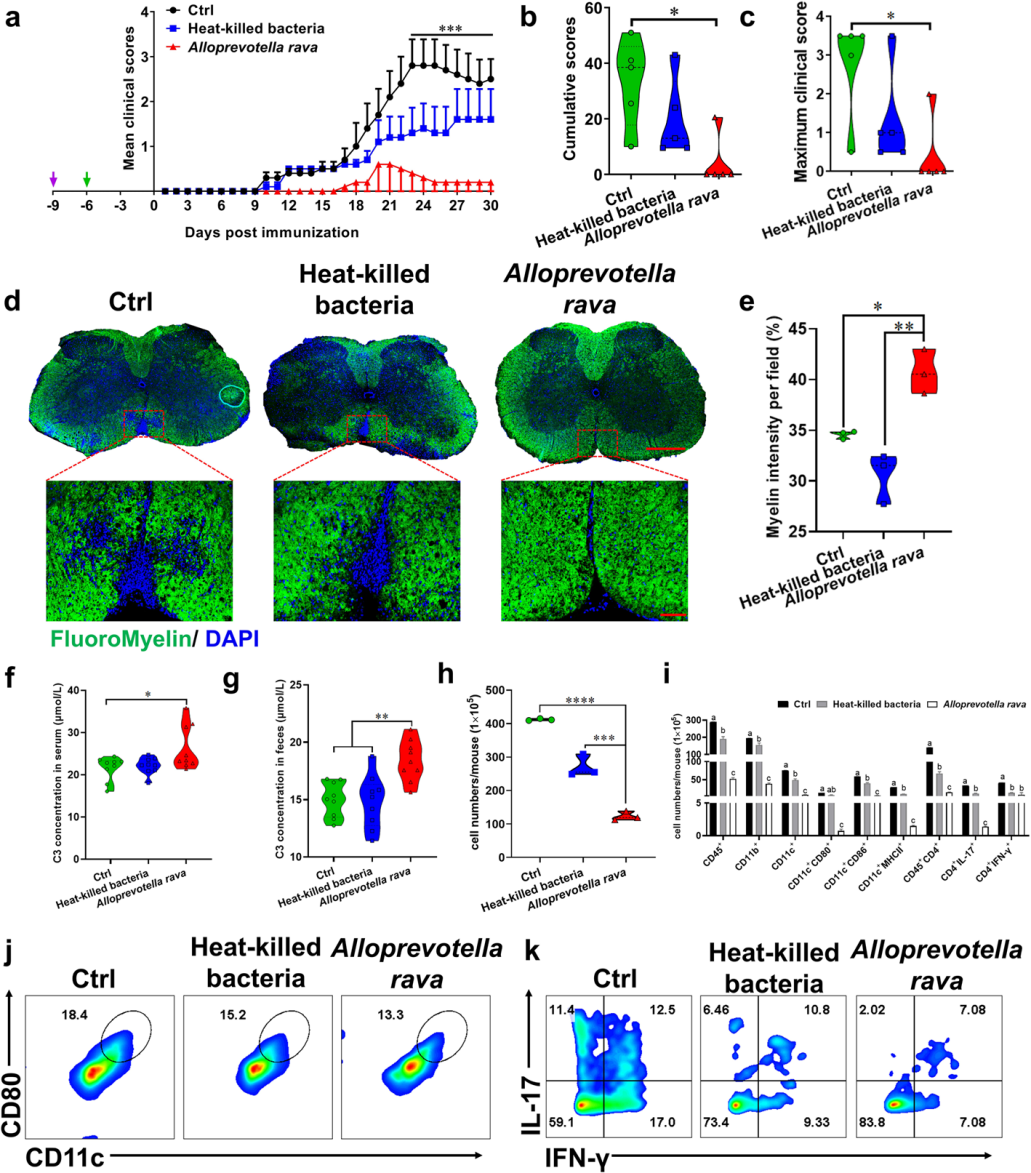
*Alloprevotella rava* colonization significantly alleviated EAE mice. C57BL/6 mice were orally administered ABX for three days followed by oral gavage of *Alloprevotella rava* or heat-killed *Alloprevotella rava* for 7 days before immunization. Immunization with MOG_35-55_ was initiated from day 0, and feces were collected from mice in each group for fecal transplantation after immunization, as indicated by arrows. **a** Disease was scored daily on a 0–5 scale. *** indicates the significant difference between Ctrl and *Alloprevotella rava* from day 23 p.i. to 30 p.i. (*p* < 0.001). **b** Cumulative scores of EAE (sum of daily clinical scores). **c** Maximum clinical score. Ctrl, Heat-killed or *Alloprevotella rava*-treated EAE mice described in (**a**) were sacrificed at day 30 p.i., and spinal cords were harvested. Spinal cords sections were analyzed by (**d**) Immunofluorescent staining of myelin sheaths. Full slice bar = 500 μm, partial slice bar = 50 μm. **e** Statistical analysis of (**d**). **f**-**g** C3 content in feces and serum of ctrl, heat-killed or *Alloprevotella rava* treated EAE mice on day 30 of immunization. **h** Total number of MNCs in the CNS. **i**-**k** The percentages and absolute numbers of various MNC subpopulations were measured by flow cytometry. Data are expressed as mean ± SEM (*n* = 5 mice in each group), *p < 0.05, **p < 0.01 and ***p < 0.001, ****p < 0.0001, determined by two-way ANOVA (a), or one-way ANOVA (b, c, e, f-i). One representative of three independent experiments is shown

EA greatly enriched genus *Alloprevotella* in the gut microbiota of EAE mice (Fig. 2c, Fig. 4b). *Alloprevotella rava* is currently known to be a species belonging to the genus *Alloprevotella* isolated from the human oral cavity [9]. We therefore invesitgated the potential role of *Alloprevotella rava* in inhibiting EAE development. Mice were administered ABX for 3 consecutive days to deplete the gut microbiota. Subsequently, sterile water, live, or heat-killed *Alloprevotella rava* (DSM 22548) was orally administered for a duration of 7 days. After *Alloprevotella rava* was colonized, immunization was started. Fresh feces were collected from mice in each group on the day of immunization, and fecal supernatants were orally administered to mice in each group every day until the end of the experiment. We analyzed the abundance of Alloprevotella rava in each mouse group across various time points and observed a decrease in *Alloprevotella rava* abundance in all mouse groups following antibiotic administration. Compared to the control mice treated with sterile water, *Alloprevotella rava* abundance was significantly increased in the live or heat-killed *Alloprevotella rava*-treated group at days 3 and 7 p.i (Figure S5a-c). Oral administration of live *Alloprevotella rava* remarkably reduced disease severity (Fig. 6a-c). Analysis of CNS tissue sections showed that colonization with *Alloprevotella rava* inhibited demyelination in EAE mice (Fig. 6d-e).

As expected, live *Alloprevotella rava* significantly increased C3 in serum and feces of EAE mice (Fig. 6f, g), along with the reduction in the total number of MNCs (Fig. 6h). Consistently, the *Alloprevotella rava*-treated group exhibited lower percentages and absolute numbers of CD45^+^ leukocytes, CD11b^+^ cells (macrophages/microglia), CD11c^+^ cells (dendritic cells), and costimulatory molecules (eg, MHC II, CD80, and CD86), as well as IFN-γ^+^ and IL-17^+^ CD4^+^ T cells (Fig. 6i-k, Figure S5d), suggesting its effects on suppressing CNS inflammation. Interestingly, we found that heat-killed *Alloprevotella rava* also appeared to ameliorate disease, without increasing C3 content in blood and feces of EAE mice (Fig. 6f, g). These findings suggest that EA can foster a beneficial microbial environment by directly stimulating the growth of *Alloprevotella rava* and C3, subsequently reducing disease severity in EAE mice.

To investigate mechanism underlying the beneficial effects of *Alloprevotella rava* on ameliorating EAE, we conducted RNA-seq analysis of spinal cords following *Alloprevotella rava* treatment. PCA analysis showed good intergroup separation and intragroup correlation in each group of samples (Figure S6a). Oral administration of live or heat-killed *Alloprevotella rava* greatly affected gene profiles (Figure S6b, c), resulting in a total of 865 genes differentially expressed between *Alloprevotella rava* and the Ctrl group, and 431 differential genes between live bacteria versus heat-killed bacteria, respectively (Figure S6d, e). The presence of live or heat-killed *Alloprevotella rava* led to distinct patterns of gene profiles (Figure S6f). Th17 differentiation-related genes were particularly down-regulated by live *Alloprevotella rava* treatment compared with the other two groups (Figure S6g, h). KEGG enrichment results indicate that Th17 differentiation pathway may mediate the ameliorative effect of *Alloprevotella rava* on EAE (Figure S6i).

### EA-enhanced *Alloprevotella rava* and C3 inhibited Th17 polarization by regulating protein acetylation in vitro

**Fig. 7:**
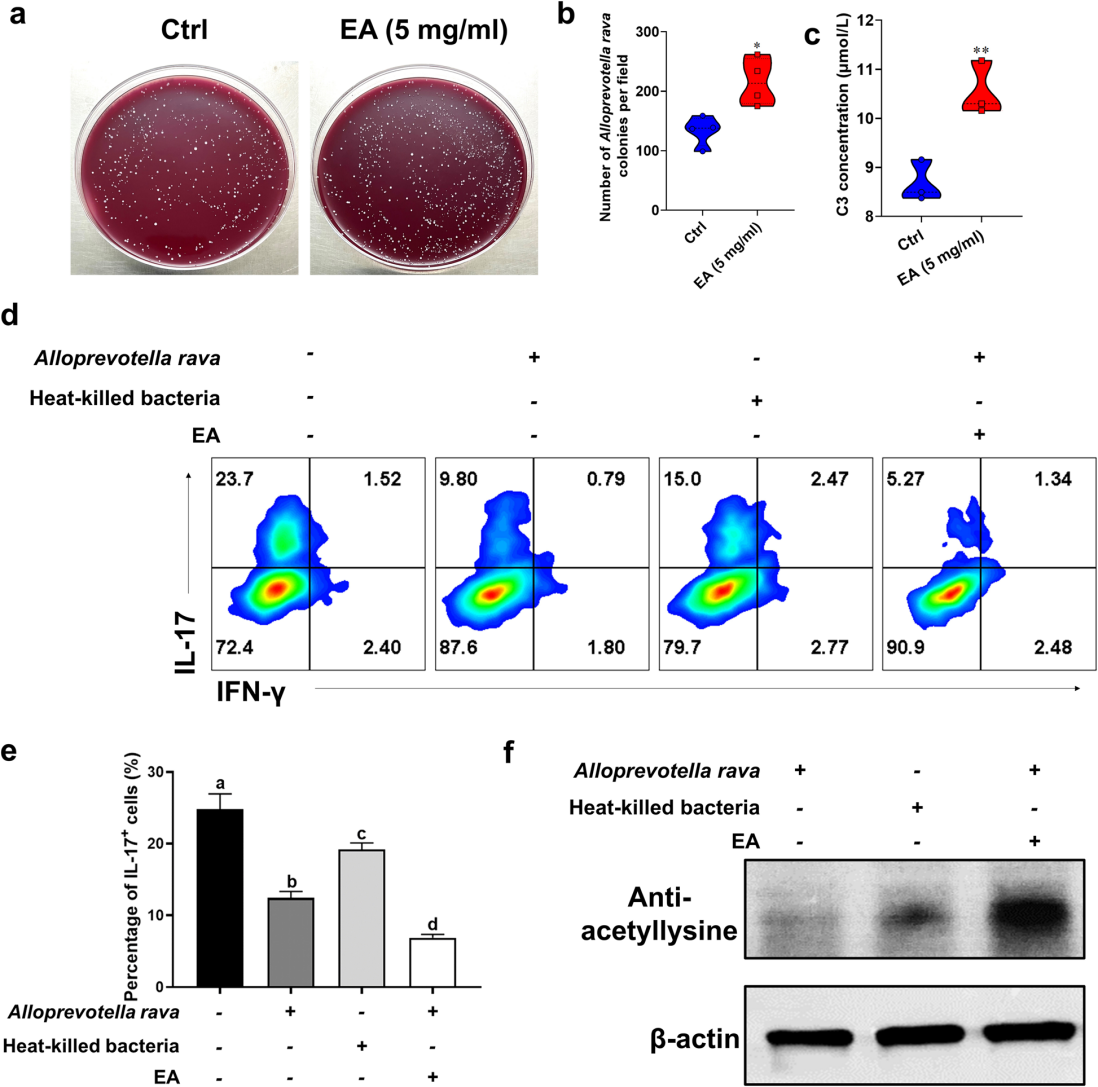
EA promoted *Alloprevotella rava* growth and inhibited Th17 polarization by regulating protein acetylation. **a** EA (5 mg/ml) co-cultured with *Alloprevotella rava *in vitro. **b** Number of colonies growing in the per field of in vitro co-cultivation of sterile water, EA, and *Alloprevotella rava*. **c** Concentration of C3 produced by in vitro co-culture of sterile water, EA, and *Alloprevotella rava*. CD4^+^ cells were isolated from spleen of naïve C57BL/6 mice and cultured under Th17 polarizing conditions for 72 h under vehicle or EA treatment. **d**-**e** CD4^+^ T cells were treated with vehicle or EA for 72 h under the Th17 condition. IL-17^+^ production was analyzed by flow cytometry. **f** CD4^+^ T cells were treated with vehicle or EA under the Th17 condition for 24 h. The effect on protein acetylation expression in Th17 cells was analyzed by Western blot. *p < 0.05, **p < 0.01 and ****p < 0.0001. The same letter represents NO significant difference between the two groups. Data are expressed as mean ± SEM (*n* = 5 each group), determined by unpaired Student’s *t*-test (b, c), and one-way ANOVA (**e**). One representative of three independent experiments is shown

The addition of EA in the culture medium significantly enhanced the growth of *Alloprevotella rava* (Fig. 7a-b), resulting in significantly elevated C3 levels compared to those observed in *Alloprevotella rava* cultured in the absence of EA (Fig. 7c). To further establish a strong link between EA-induced growth promotion of *Alloprevotella rava*, C3, and histone acetylation, we administered the cell free supernatant of *Alloprevotella rava* cultured with EA to Th17 cells. Notably, the supernatants from *Alloprevotella rava* co-cultured with EA were potent in inhibiting polarization of Th17 cells (Fig. 7d,e). C3 is a known HDAC inhibitor [23] that could induce acetylation of histones [24]. As expected, we found that the supernatant of *Alloprevotella rava* co-cultures with EA significantly enhanced the protein acetylation under Th17 polarization conditions (Fig. 7f). These results indicate that EA could enrich *Alloprevotella rava* and produce C3, thereby modulating Th17 cell acetylation.

### Oral administration of C3 alleviated EAE

**Fig. 8:**
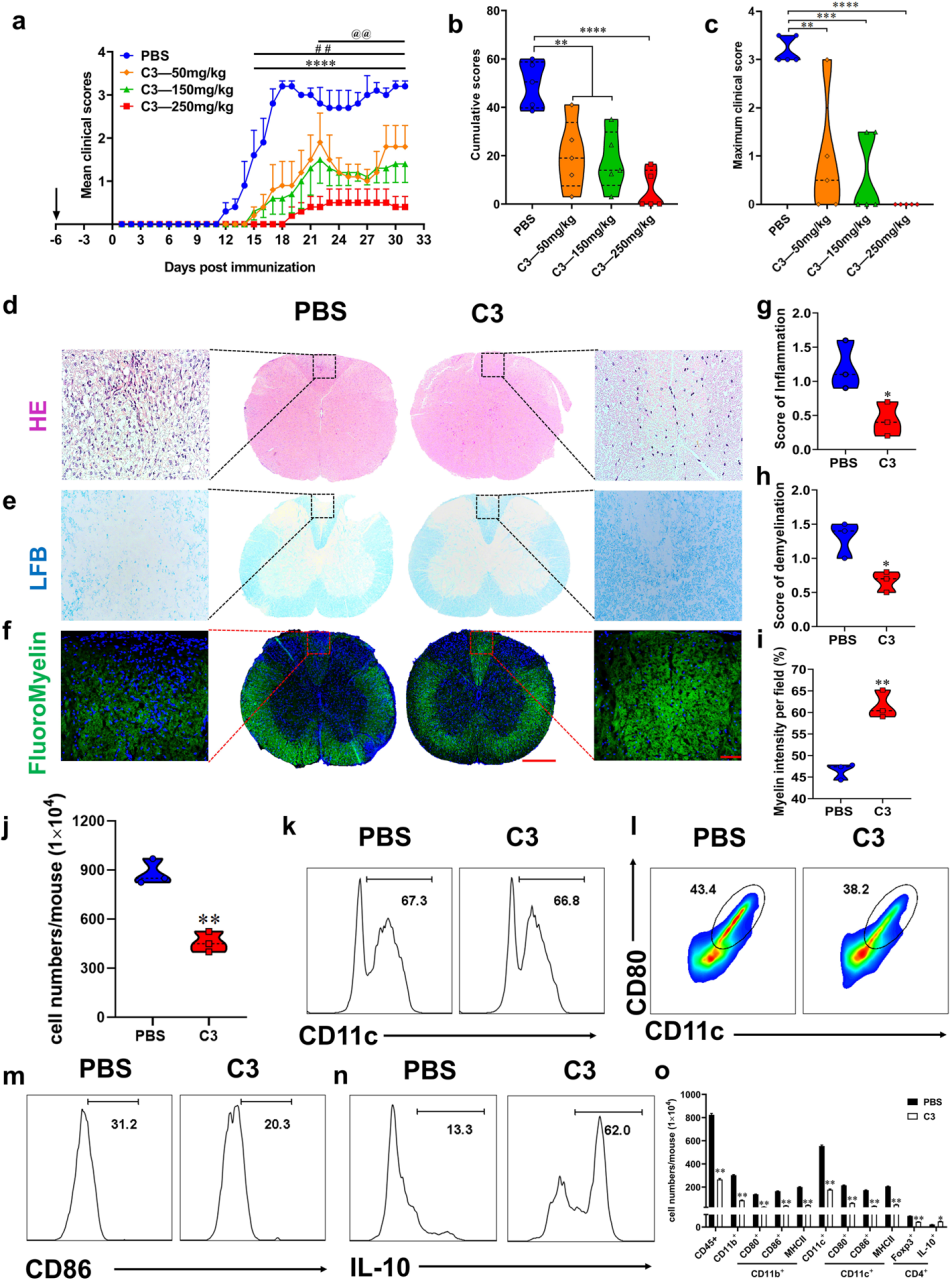
Oral C3 treatment remarkably alleviated EAE mice. C57BL/6 mice were orally administrated with PBS and different doses of C3 7 days before immunization, and immunized with MOG_35-55_ from day 0 as indicated by the arrow. **a** Disease was scored daily on a 0–5 scale. @@ indicates the significant difference between PBS and C3 (50 mg/kg) from day 22 p.i. to 31 p.i. (*p* < 0.01). ## indicates the significant difference between PBS and C3 (150 mg/kg) from day 16 p.i. to 33 p.i. (*p* < 0.01). **** indicates the significant difference between PBS and C3 (250 mg/kg) from day 16 p.i. to 33 p.i. (*p* < 0.0001). **b** Cumulative scores of EAE (sum of daily clinical scores). **c** Maximum clinical score. C3- or PBS-treated control EAE mice described in (a) were sacrificed at day 30 p.i., and spinal cords were harvested. Spinal cords sections were analyzed by (**d**-**f**) Luxol fast blue (LFB, for degree of demyelination), haematoxylin and eosin (H&E, for inflammation), and immunofluorescent staining of myelin sheaths. Full slice bar = 500 μm, partial slice bar = 50 μm. **g-i** Statistical analysis of (d-f). **j**) Total number of MNCs in the CNS. **k-o** The percentages and absolute numbers of various MNC subpopulations were measured by flow cytometry. Data are expressed as mean ± SEM (*n* = 5 mice in each group), *p < 0.05, **p < 0.01 and ***p < 0.001, ****p < 0.0001, determined by two-way ANOVA (a), one-way ANOVA (b, c), or unpaired Student’s *t*-test (g-j), or Mann Whitney test (o). One representative of three independent experiments is shown

Our results indicate that C3, the most abundant metabolite in the EA group, exhibits mitigating effects on the progression of EAE. Here, we administered varying dosages of C3 to EAE mice to investigate its direct effects. Compared to the PBS-treated group, C3 significantly reduced mean clinical scores (Fig. 8a), cumulative scores (Fig. 8b), and peak clinical scores (Fig. 8c) in a dose-dependent manner, led to a remarkably reduction in inflammation and demyelination (Fig. 8d-i).

Moreover, C3 dramatically reduced the total number of infiltrating cells (Fig. 8j), the percentage and an absolute number of CD45^+^ leukocytes, co-stimulatory molecules (e.g., MHC class II, CD80, and CD86) of CD11b^+^ cells (macrophages/microglia), CD11c^+^ cells (dendritic cells) and nTreg cells (CD4^+^Foxp3^+^). By contrast, C3 increased the percentage and absolute number of CD4^+^ IL-10^+^, suggesting increased IL-10 secretion by iTreg cells [25] (Fig. 8k-o, Figure S7a-f). These results indicate that C3 effectively inhibited CNS inflammation.

**Fig. 9:**
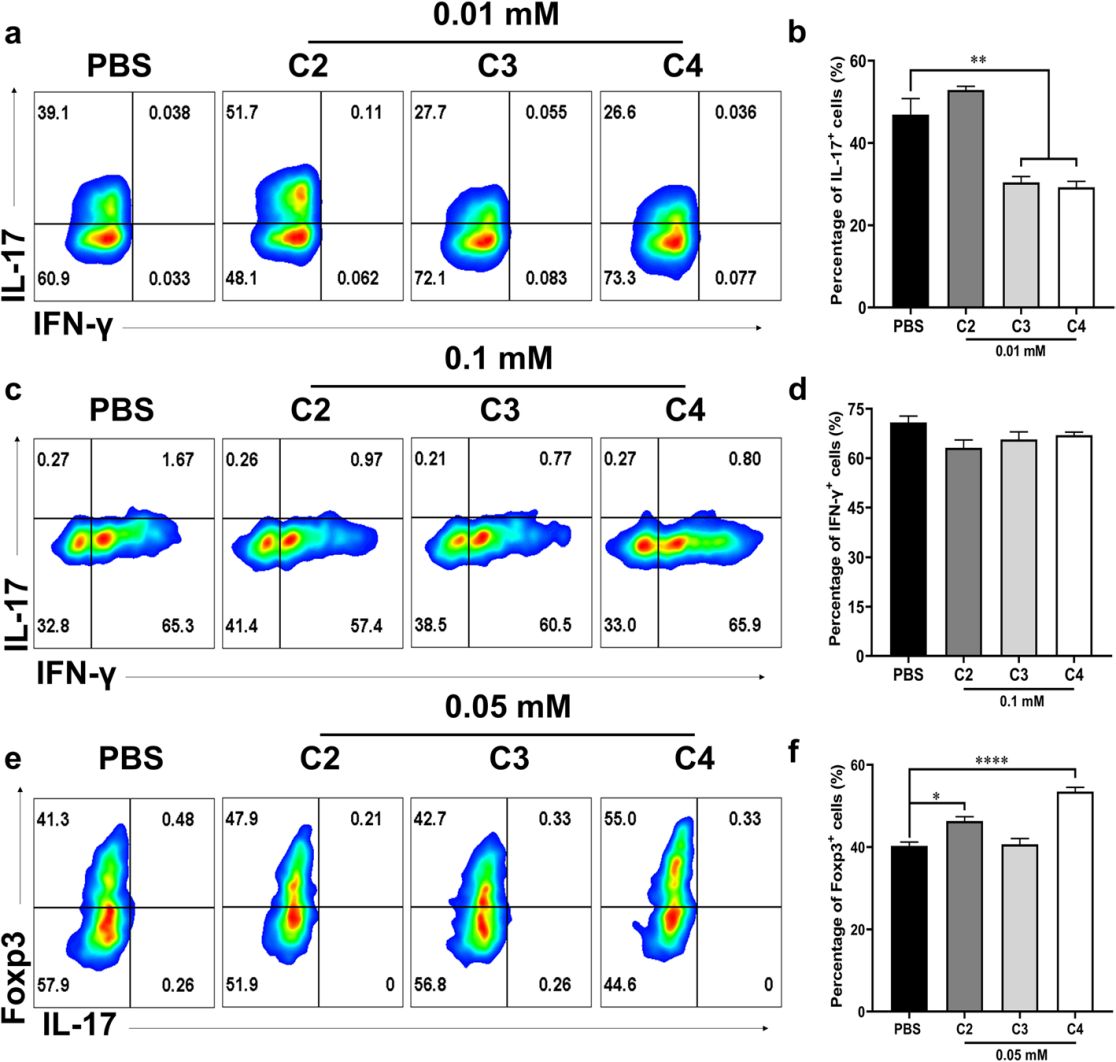
Effects of SCFAs on Th1, Th17, or Treg differentiation in vitro. CD4^+^ cells were isolated from the spleens of naïve C57BL/6 mice and cultured under Th17, Th1, and Treg polarizing conditions and of different treatment concentrations of C2, C3 or C4 for 72 h. **a** CD4^+^ T cells were treated with C2, C3 or C4 (0.01 mM) for 72 h under Th17 polarizig conditions. IL-17^+^ production was analyzed by flow cytometry. **b** Statistical analysis of (a). **c** CD4^+^ T cells were treated with C2, C3 or C4 (0.1 mM) for 72 h under the Th1 condition. IFN-γ^+^ production was analyzed by flow cytometry. **d** Statistical analysis of (c). **e** CD4^+^ T cells were treated with C2, C3 or C4 (0.05 mM) for 72 h under the Treg condition. Foxp3^+^ production was analyzed by flow cytometry. **f** Statistical analysis of (**e**). Data are expressed as mean ± SEM (*n* = 3 each group), *p < 0.05, **p < 0.01 and ****p < 0.0001, determined by unpaired one-way ANOVA (b, d, f). One representative of three independent experiments is shown

In addition, we also explored how other SCFAs [acetate (C2), C3, or butyrate (C4)] could influence the Th1, Th17, or Treg differentiation in vitro. C3 and C4 had comparable inhibitory effects on Th17 polarization (Fig. 9a,b), but had no effect on Th1 cells (Fig. 9c-d). C2 had some promoting effect on Treg cells, while C4 presented a significant promoting effect (Fig. 9e-f). C3 had a specific inhibitory effect on Th17 cells at a low dose.

### C3 inhibited Th17 polarization by modulating acetylation via inhibiting HDACs

**Fig. 10:**
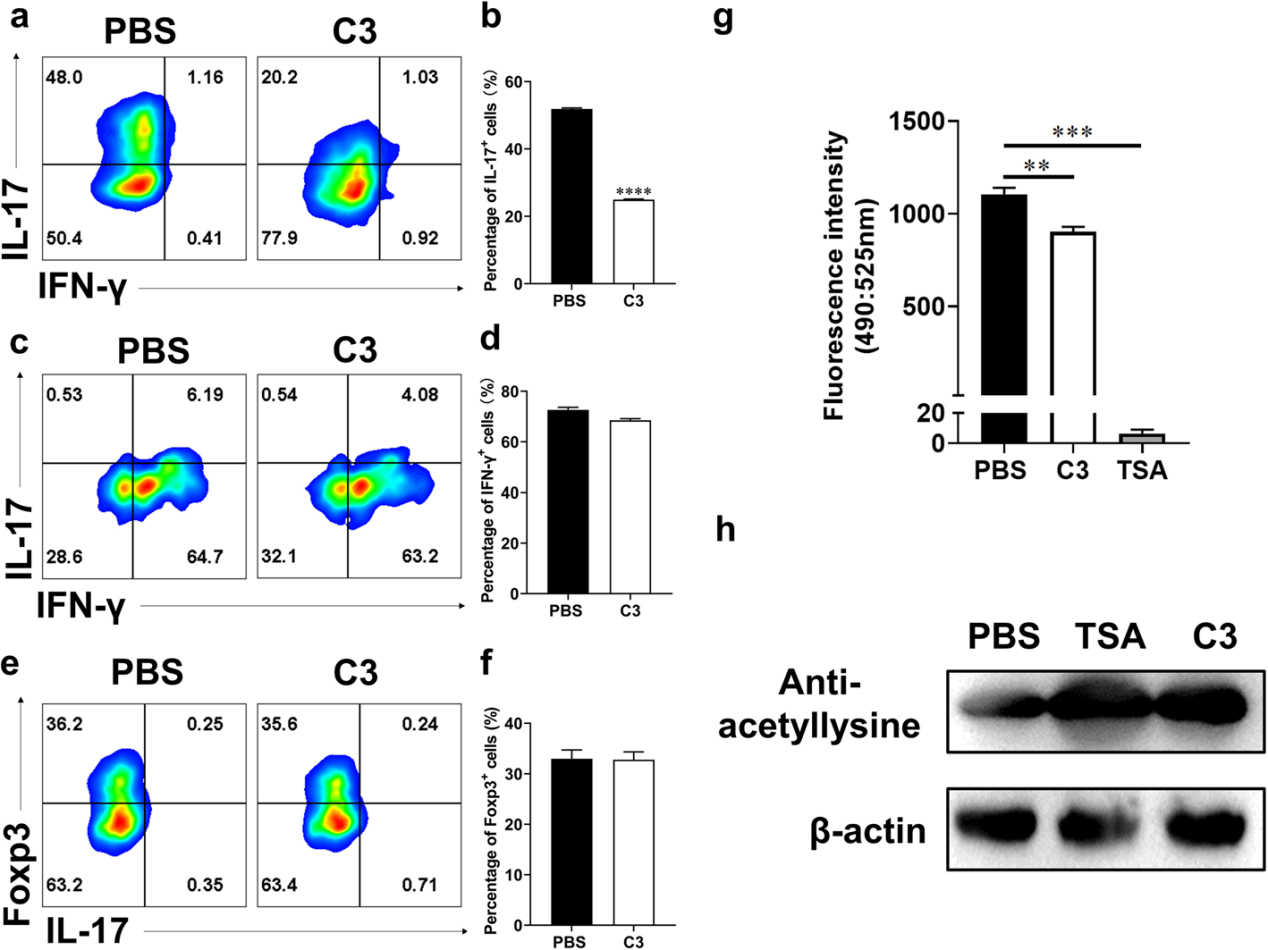
C3 inhibited Th17 polarization by modulating acetylation via inhibiting HDACs. CD4^+^ cells were isolated from spleen of naïve C57BL/6 mice and cultured under Th17 polarizing conditions under treatment of different concentrations of C3 for 3 days. **a**-**b** CD4^+^ T cells were treated with C3 (0.01 mM) for 72 h under the Th17 condition. IL-17^+^ production was analyzed by flow cytometry. **c**-**d** CD4^+^ T cells were treated with C3 (0.1 mM) for 72 h under the Th1 condition. IFN-γ^+^ production was analyzed by flow cytometry. **e**–**f** CD4^+^ T cells were treated with C3 (0.05 mM) for 72 h under the Treg condition. Foxp3^+^ production was analyzed by flow cytometry. **g** CD4^+^ T cells were cultured under Th17 polarizing condition, in the presence or absence of C3 (1 mM) or TSA (0.5 μM), collected at 24 h, and analyzed for HDAC activity using an HDAC activity assay kit at a fluorescence intensity of excitation/emission (490/525 nm). **h** CD4^+^ T cells were cultured as described in **g**. The effect of C3 on protein acetylation expression in Th17 cells was analyzed by Western blot. Data are expressed as mean ± SEM (*n* = 3 each group), **p < 0.01 and ***p < 0.001, ****p < 0.0001, determined by unpaired Student’s *t*-test (b, d, f), or determined by one-way ANOVA (g). One representative of three independent experiments is shown

Autoreactive T cells and monocytes traverse the blood–brain barrier to enter the CNS, where they become activated by microglia and macrophages. This activation leads to inflammation and promotes demyelination [26]. To test whether C3 could modulate the CD4^+^ T cell differentiation, we polarized Th1, Th17, and Treg cells in the presence or absence of C3. Cell viability assays showed that C3 was not toxic to T cells up to 1 mM (Figure S8a). The percentage of Th17 cells expressing CD4^+^ and IL-17^+^ markers was significantly decreased by C3 treatment, compared to the PBS control. Even at a low concentration of 0.01 mM, C3 demonstrated a pronounced inhibitory effect (Fig. 10a-b, Figure S8b). We also analyzed the effects of C3 treatment on Th1 (CD4^+^ IFN-γ^+^) or Treg (CD4^+^ Foxp3^+^) cell differentiation in vitro. C3 (1 mM) failed to decrease the percentage of Th1 cells (Fig. 10c-d, Figure S8c). C3 (0.05 mM) had no impact on Treg cell percentage (Fig. 10e-f, Figure S8d). These results indicated that Th17 cells were more sensitive to a low-dose C3 treatment (0.01 mM) than that of other T helper cell subsets.

HDAC is the target of SCFAs, and inhibitory effect on HDACs is essential for maintaining immune homeostasis [27]. We thus investigated whether the inhibitory effect of C3 on Th17 development was attributed to the inhibition of HDAC activity. CD4^+^ T cells were cultured under Th17 polarizing condition and were treated with or without C3 for 24 h. HDAC inhibitor trichostatin A (TSA) was used as the positive control. Our findings indicate that C3, albeit to a lesser extent compared to TSA, attenuated HDAC activity in Th17 cells (Fig. 10g) and C3 indeed enhanced protein acetylation under Th17 polarizing conditions (Fig. 10h). These results indicate that C3 could suppress Th17 cell differentiation by inhibiting HDAC activity and modulating protein acetylation levels.

## Discussion

The pathogenesis of MS is complex and has not been completely explained. Increasing evidence has indicated that gut microbiota plays a critical role in the development of MS, and specific gut microbiota may be associated with disease alleviation [28, 29]. Prebiotics can target feeding gut microbiota and selectively promote beneficial bacteria growth and metabolism, thereby improving disease [30]. Based on the comprehensive in vivo and in vitro investigations, the present study establishes that dietary polyphenol EA mitigated the development and progression of EAE disease via the microbiota-metabolites-immunity axis, and offers compelling evidence on the mediating role of *Alloprevotella rava* and propionate metabolism in the benefits of EA on EAE alleviation. Our study provides novel insights in the EA as a potential prebiotic for prevention and treatments of MS.

Our previous study found that gut microbiota-mediated the ameliorative effect of oral pomegranate peel extracts (PPE) on EAE, with EA identified as the primary constituent of PPE [31]. Many studies focusing on EA metabolism have reported that EA is rarely detected in the systemic circulation, due to its low oral bioavailability. URA is the major reported metabolite of EA [18]. Our findings indicate that EA, as a precursor of URA, exhibited a more pronounced therapeutic effect than URA itself in mitigating EAE, suggesting the existence of a URA-independent mechanism of action. Tasaki et al. conducted a 90-day sub-chronic study of EA in F344 rats, suggesting that the no observed-toxic-effect level (NOTEL) was 5% for males (3011 mg/kg/d) and for females (3254 mg/kg/d) [32]. EA has been used as a food additive in Japan [33], and is naturally abundant in vegetables, fruits, and nuts that are core component of the MedDiet, a dietary pattern has shown to reduce the disease severity of MS patients [15]. In the present study, we demonstrated that oral EA could effectively reduce the development of EAE, inhibited DC/microglia/macrophage activation, and reduce Th17 cell differentiation in vivo. The effective dosage of EA (25 mg/kg/d) is ∼120 or 130 times lower than the dose showing NOEL (3254 mg/kg/d) or NOAEL (3254 mg/kg/d), reinforce its great potential to be used for MS treatments.

EA is a polyphenol that accumulates in large amounts in the gastrointestinal tract and needs to be degraded by gut microbiota in the colonic site [34]. To investigate the effect of EA treatment on the gut microbial composition in EAE mice, we collected fecal samples at the peak time point of clinical disease (17 ~ 19 days p.i.). EA caused a significant increase in the relative abundance of *Alloprevotella* and increased fecal levels of 14 metabolites, with C3 being the most significantly up-regulated. We found that EA-constructed fecal bacteria for 42 days had significant improvement in EAE, and that the therapeutic effect of EA on EAE was substantially weakened in gut microbiota depleted mice, confirming the essential role of gut microbiota involved in the therapeutic effect of EA on EAE.

Most importantly, our study identified *Alloprevotella rava* as the core bacteria responsible for C3 production and EAE alleviation, which to the best of knowledge has not been reported before. *Alloprevotella* is a reported SCFAs producer [21]. *Alloprevotella rava* produces large amounts of succinic acid as an end product of its metabolism [9] and, in turn, propionate can be produced via the succinic acid pathway [12]. We verified in vitro that EA could directly promote *Alloprevotella rava* growth, thereby promoting C3 production. *Alloprevotella rava* co-cultured with EA successfully inhibited Th17 polarization by promoting protein acetylation in cell models. Moreover, intestinal colonization of live *Alloprevotella rava* in EAE mice significantly promoted C3 production and ameliorated the development of the disease. Besides, we found that heat-killed *Alloprevotella rava* also to a great extent alleviated EAE symptoms. The heat-killed *Alloprevotella rava* may play a beneficial role as metazoan through bacterial components such as peptidoglycan and teichoic acid, as well as metabolites such as vitamins, lipids, and short-chain fatty acids. Heat-killed *Lactobacillus rhamnosus* IDCC 3201 tyndallizate (RHT3201) relieved atopic dermatitis [35]. Heat-killed Bifidobacterium bifidum MIMBb75 (SYN-HI-001) improved irritable bowel syndrome [36]. Our results suggest that both live *Alloprevotella rava* and heat-killed *Alloprevotella rava* might be potential therapeutic agents that can be developed into functional probiotics and postbiotics to combat CNS autoimmune diseases.

In addition, oral EA remarkably reduced the relative abundance of *Akkermansia* in EAE mice, suggesting a potential alternative target of EA that may ameliorate MS. *Akkermansia muciniphila* has been reported to exhibit health-promoting effects in diabetic, obesity and its associated metabolic diseases [37, 38]. However, Sushrut Jangi et al., found that *Akkermansia muciniphila* was increased in MS patients compared with healthy controls, in the USA population and that oral probiotics (*Lactobacillus*, *Bifidobacterium*, and *Streptococcus*) treatment reduced the abundance of *Akkermansia* in MS patients [39, 40]. Other studies also found that MS patients had higher abundances of *Akkermansia*, *Streptococcus*, *Methanobrevibacter* compared with healthy controls [39, 41, 42], in line with our results.

SCFAs are closely related to immune and metabolic functions. Research regarding butyrate on MS shows that a Clostridia strains mixture derived from human gut aided in the prevention of EAE, but the therapeutic effect was not statistically significant [43]. Our study demonstrates the core role of C3 promoted by the EA-enriched *Alloprevotella rava* on EAE treatment. In line with our data, Duscha et al. found that both sera and feces from MS patients showed obviously reduced amounts of propionic acid and reduced relative abundance of SCFAs producing bacteria, and that C3 supplementation was able to activate Treg function and improve immune dysregulation in MS patients, thereby improving MS [44]. A study on patients with inflammatory bowel disease found that SCFAs, metabolites of gut microbiota, in particular propionate, regulate IL-17 and IL-22 production by human intestinal γδ T lymphocytes by inhibiting HDAC activity [45].

Mechanistically, we characterized the mechanism of C3 action on Th17 cells in MS/EAE, which has not been previously reported. C3 is a known ligand for G protein-coupled receptors (GPCRs) and inhibitors of HDAC [27]. Healthy gut microbiota producing SCFAs can promote Treg cell production in a GPR43-dependent manner and induce histone H3 acetylation [46]. While Park et al. concluded that GPR41 and GPR43 were unnecessary for SCFAs to affect the process of T cell differentiation and that the effect of SCFAs on T cells was achieved by inhibiting HDAC activity [47]. C3 is an endogenous inhibitor of HDAC and can inhibit HDAC-induced deacetylation within histones [48]. Our study showed that C3 dose-dependently ameliorated EAE, inhibited the activity of HDAC and increased protein acetylation levels in Th17 cells. HDAC (Sirt1) can deacetylate retinoid receptor-associated orphan receptor γ (RORγt), resulting in the attenuation of the transcriptional activity of RORγt once acetylated and subsequently inhibiting the induction of IL-17A and the differentiation of Th17 cells [49, 50]. RORγt is a key factor regulating Th17 differentiation and induces the expression of IL-17A and IL-17F [51, 52]. EA and *Alloprevotella rava* significantly decreased IL-17A expression in spinal cord of EAE mice. These results consistently suggest that C3, as an endogenous inhibitor of HDACs, may elevate RORγt acetylation levels in Th17 cells, suppress their transcription to produce IL-17A, inhibit polarization of Th17 cells, ultimately hindering the development of EAE.

Furthermore, C3 is a typical microbial metabolite and a kind of postbiotic that has been shown to multiple probiotic effects [53]. C3 as a crucial factor contributed to the therapeutic effectivenesss of EA and has high affinity with HDAC, highlighting its great potentials as a low-toxicity drug candidate for the treatment of MS. Previous studies reported that beneficial effects of one postbiotic is limited, and the use of prebiotics or symbiotics can better promote host health by directly regulating gut microbiota homeostasis to produce more metabolites [54]. In our study, we found that the effective dose of C3 (50–250 mg/kg/d) for EAE mice is 2–tenfold higher than that of EA (25 mg/kg/d). Therefore, we proposed that advanced combination therapies with EA-containing diets as prebiotics, SCFA-producing probiotics, and/or postbiotics such as C3 may effectively achieve the treatment of MS.

## Conclusions

**Fig. 11:**
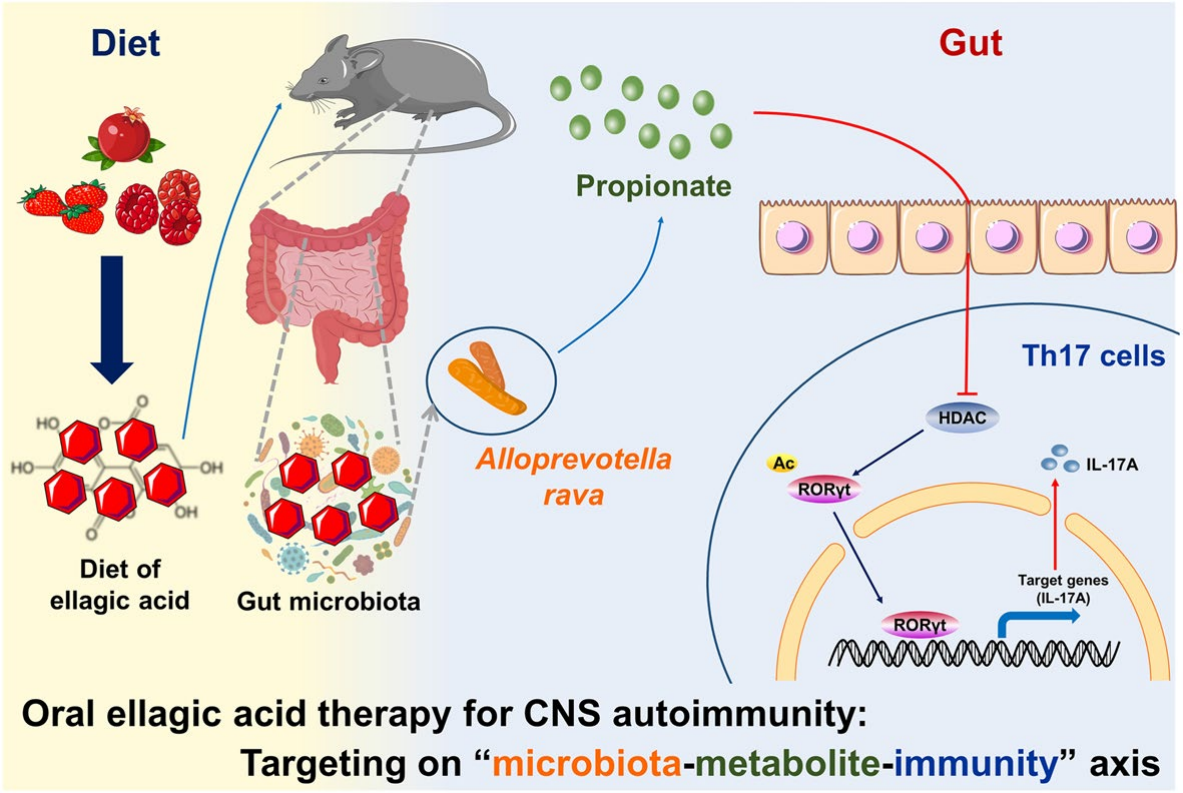
The beneficial effects of oral EA on EAE through interaction with the gut microbiome. EA enriched in fruits and nuts can directly modulate gut bacteria in EAE mice, and significantly increase the abundance of *Alloprevotella* at the genus levels. Correlation analysis showed that *Alloprevotella* was positively correlated with the content of C3, which was most significantly up-regulated at the metabolic level. EA directly promoted *Alloprevotella rava* (DSM 22548) growth and C3 production in vitro, and the supernatants of EA and *Alloprevotella rava* co-culture inhibited Th17 differentiation by modulating protein acetylation. Alleviation of EAE development and maintenance of homeostasis of the intestinal environment following *Alloprevotella rava* colonization may be attributed to an increase in C3. Mechanistically, C3 can inhibit HDAC activity, which possibly through up-regulation of RORγt acetylation and inhibition of RORγt transcriptional activity to produce IL-17A, thereby inhibiting Th17 cell differentiation and ameliorate EAE

This study explored the therapeutic effects and mechanisms of action of EA on EAE via a microbiota-metabolites-immunity axis (Fig. 11). We introduce novel concepts, suggesting that EA serves as an economical next-generation prebiotic (NGP), facilitating the proliferation of probiotics and mitigating the symptoms of MS and other autoimmune disorders. While our present study provides compelling evidences, future research efforts aim to continuously explore the mechanism underlying EA-regulated *Alloprevotella rava* and C3 production. Additional clarification is required to understand the direct impact of C3 on HDAC inhibition. What dosages would be suitable for MS patients? Does C3 exert any influence on gut microbiota composition in humans? These outstanding questions need to be addressed further. Moreover, long-term side effects of EA or C3 interventions should be thoroughly investigated. It should also be noted that while the MedDiet may seem appealing, nut allergies are prevalent, and thus, this diet may not be suitable for all patient populations, further highlighting the great need of novel gut microbiota-targeted therapy, like EA and C3.

### Supplementary Information

**Supplementary Material 1.**

**Supplementary Material 2.**

**Supplementary Material 3.**

**Supplementary Material 4.**

**Supplementary Material 5.**

## Data Availability

The authors confirm that all data (including metabolomics raw data) supporting the findings of this study are available within the paper and its Supplementary material. 16S rRNA microbial sequencing analysis data are deposited in the NCBI Sequence Read Archive (SRA) under accession number PRJNA751486.

## Abbreviations

ABX: Antibiotic
C2: Acetate
C3: Propionate
C4: Butyrate
CNS: Central nervous system
EA: Ellagic acid
EAE: Experimental autoimmune encephalomyelitis
GO: Gene ontology enrichment analysis
GPCRs: G protein-coupled receptors
HDAC: Histone deacetylase
KEGG: Kyoto Encyclopedia of Genes and Genomes
MBP: Myelin basic protein
MedDiet: Mediterranean diet
MNCs: Mononuclear cells
MS: Multiple sclerosis
NGP: Next-generation prebiotic
NOTEL: No observed-toxic-effect level
NOAEL: No observed-adverse-effect level
OTUs: Operational taxonomic units
PCA: Principal component analysis
PCoA: Principal Co-ordinates Analysis
PPE: Pomegranate peel extracts
RORγt: Retinoid-relatedorphanreceptorgammat
SCFAs: Short-chain fatty acids
Th17: T helper 17
TSA: Trichostatin A
URA: Urolithin A
