# Supplementary material for: Dietary ellagic acid therapy for CNS autoimmunity: Targeting on Alloprevotella rava and propionate metabolism
Source: Microbiome. 2024 Jun 24;12:114. doi: 10.1186/s40168-024-01819-8 (PMC11194905; doi:10.1186/s40168-024-01819-8)
Supplement: Supplementary file 2 — Supplementary Material 1. [file 40168_2024_1819_MOESM1_ESM.docx]

**Supplementary Information for**

**Supplemental Figure Legends**

**
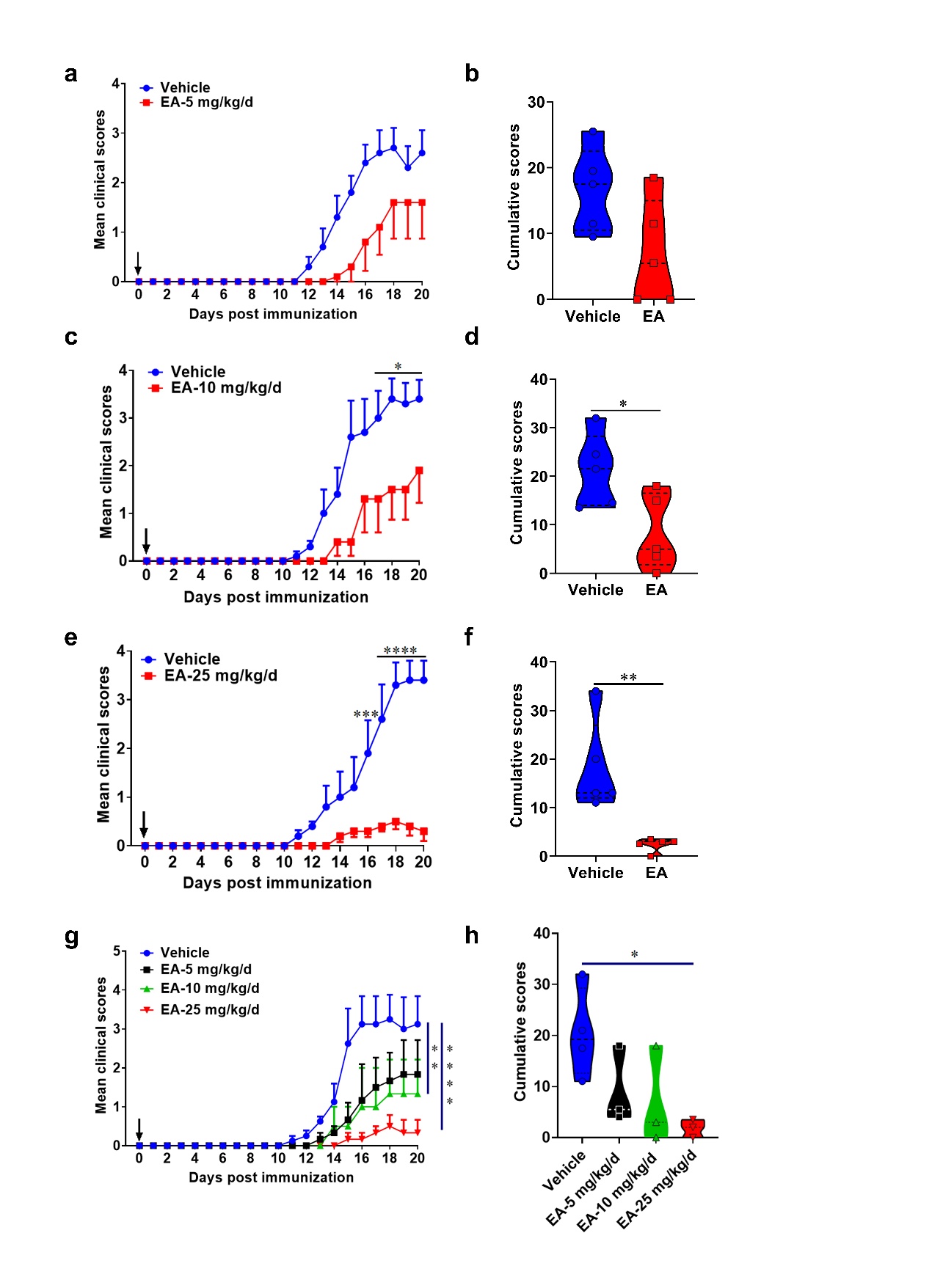
**

**Figure S1. Ameliorative effects of EA on EAE were dose-dependent.** Female, 6–8-week-old C57BL/6 mice were oral gavaged with vehicle or different doses of EA (5, 10, 25 mg/kg) on the day of EAE induction. (a, c, e, and g) Disease was scored daily on a 0-5 scale. (b, d, f, and h) Cumulative scores of EAE (sum of daily clinical scores). Data are expressed as mean ± SEM (*n* =3-5 mice in each group), **p* < 0.05, ***p* < 0.01, and *****p* < 0.0001, determined by two-way ANOVA (a, c, e, g), one-way ANOVA (h), or unpaired Student’s *t*-test (b, d, f). One representative of three independent experiments is shown.


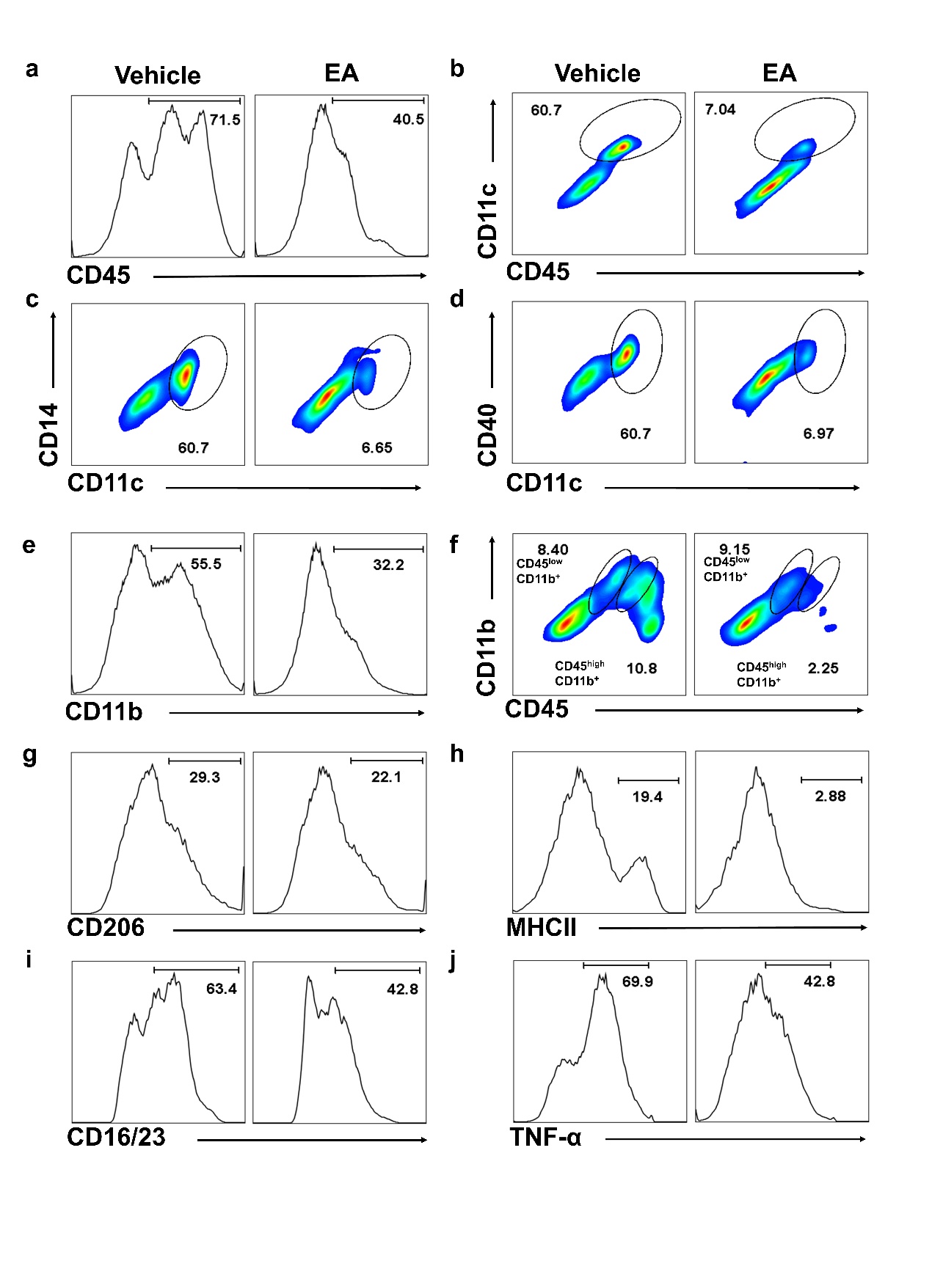


**Figure S2. EA inhibited the activation of dendritic cells and macrophages/microglia in the CNS.** Mice were orally treated with vehicle or EA (25 mg/kg) daily, starting from the day of EAE induction, and sacrificed at day 30 p.i. Spinal cords and brain were collected and MNCs were separated (*n* = 5 mice in each group). Percentages of (a) CD45^+^, (b) CD11c^+^, (c) CD11c^+^ CD14^+^, (d) CD11c^+^ CD40^+^, (e) CD11b^+^ (f) CD45^high^ CD11b^+^ and CD45^low^ CD11b^+^, (g) CD11b^+^ CD206^+^, (h) CD11b^+^ MHCII^+^, (i) CD11b^+^ CD16/32^+^, (j) CD11b^+^ TNF-α^+^ cells were assessed by flow cytometry.


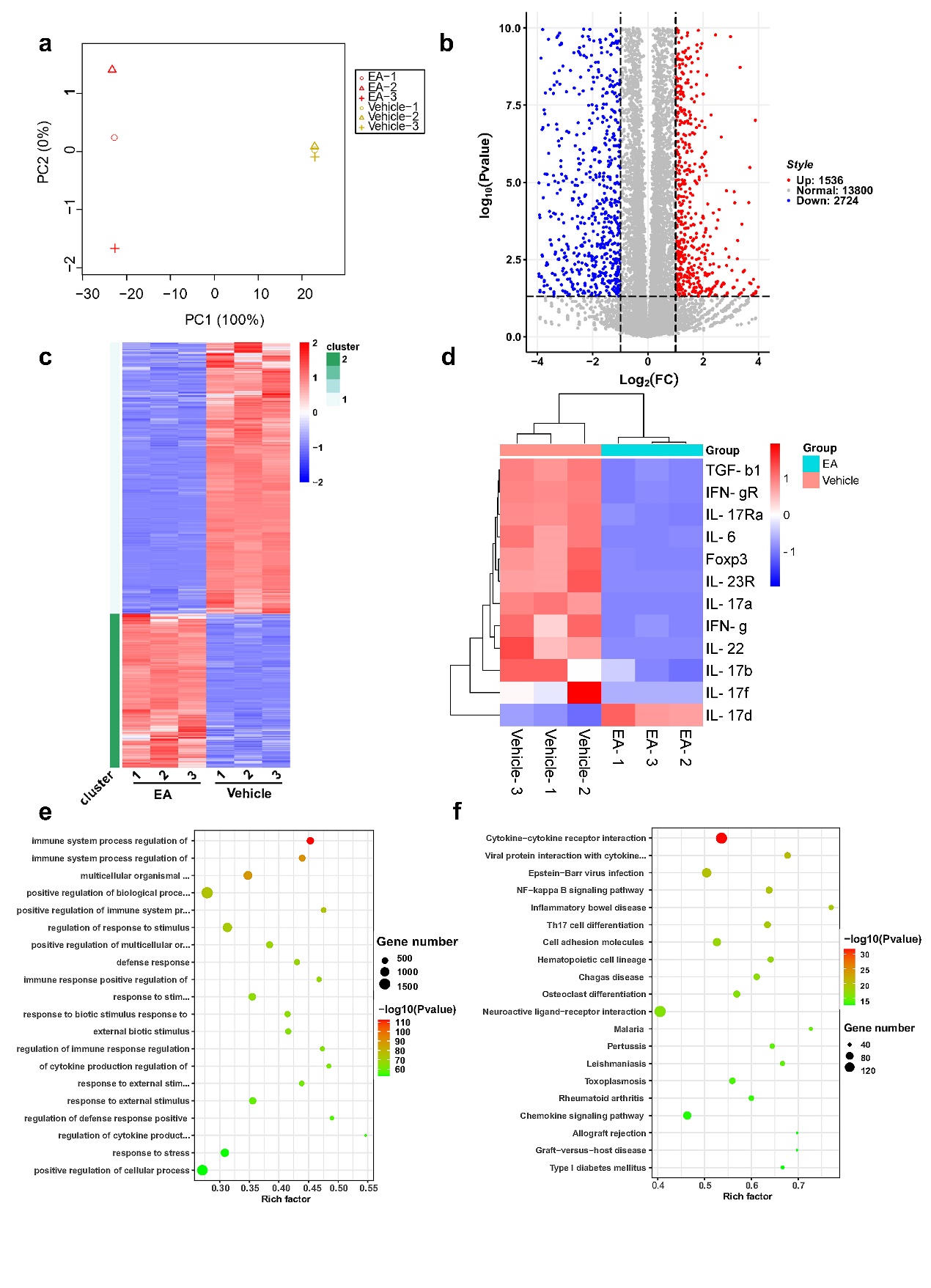


**Figure S3. EA alleviated EAE by inhibiting the Th17 cell differentiation pathway.** (a) PCA analysis between Vehicle and EA groups. (b) Volcano plot analysis of differentially expressed genes between Vehicle and EA groups. (c) Heatmap analysis of differentially expressed genes between Vehicle and EA groups. (d) Heatmap analysis of Th17 differentiation-related genes between Vehicle and EA groups. (e) Gene Ontology (GO) analysis of significantly regulated genes between Vehicle and EA treatment groups. (f) Kyoto Encyclopedia of Genes and Genomes (KEGG) analysis of significantly regulated genes between Vehicle and EA treatment groups.


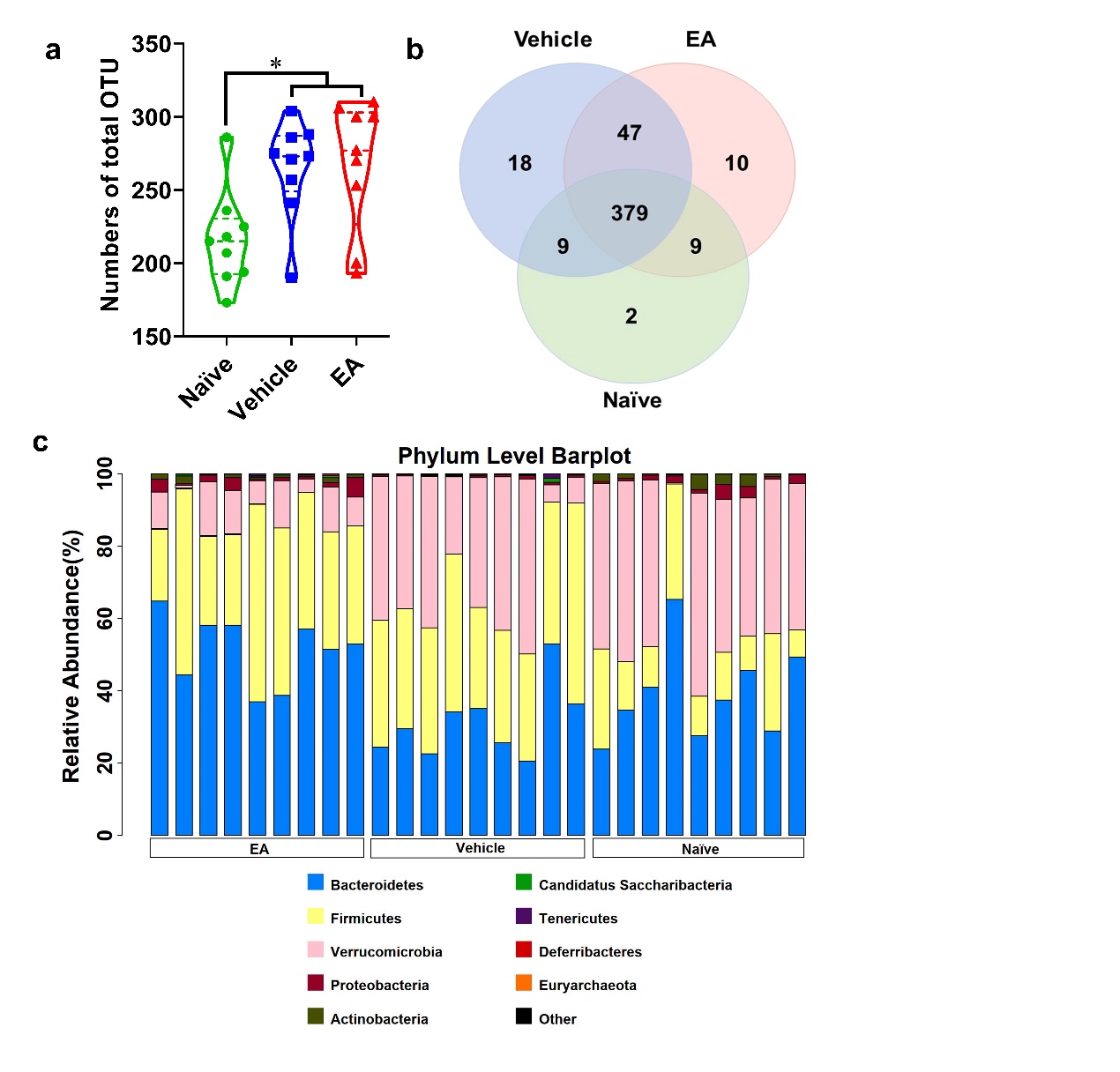


**Figure S4. EA treatment altered the gut microbial composition in EAE mice.** 16S rRNA sequencing were performed on stool samples from naïve, vehicle- and EA-treated groups (*n* = 9 each group). (a) Total OTU counts of gut microbiota. (b) Venn diagram of the three groups. (c) Bar plot of composition and relative abundance of phylum levels in the gut microbiota of the three groups. Data are expressed as mean ± SEM, **p* < 0.05, determined by two-way ANOVA (b).


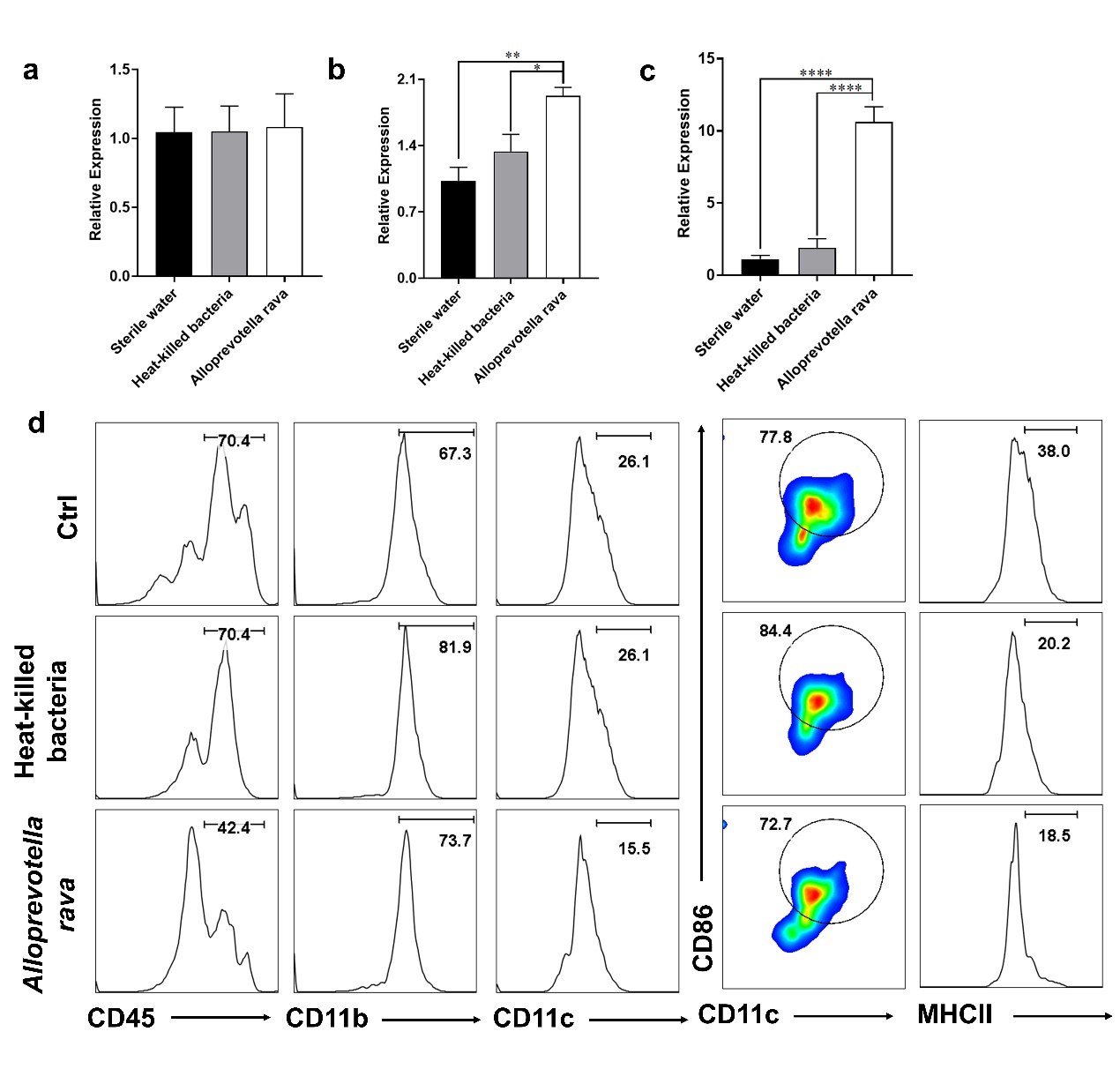


**Figure S5. EA promoted *Alloprevotella rava* growth and *Alloprevotella rava* suppressed CNS inflammation.** (a-c) Relative expression of *Alloprevotella rava* in feces of mice after antibiotic treatment and on days 3 and 7 of intragastric administration of *Alloprevotella rava*. (d) CD45^+^, CD11b^+^, CD11c^+^, CD80^+^, CD86^+^, MHCII^+^ cells were assessed by flow cytometry. Data are expressed as mean ± SEM, **p* < 0.05, ***p* < 0.01 and *****p* < 0.0001, determined by two-way ANOVA (a-c). One representative of three independent experiments is shown.


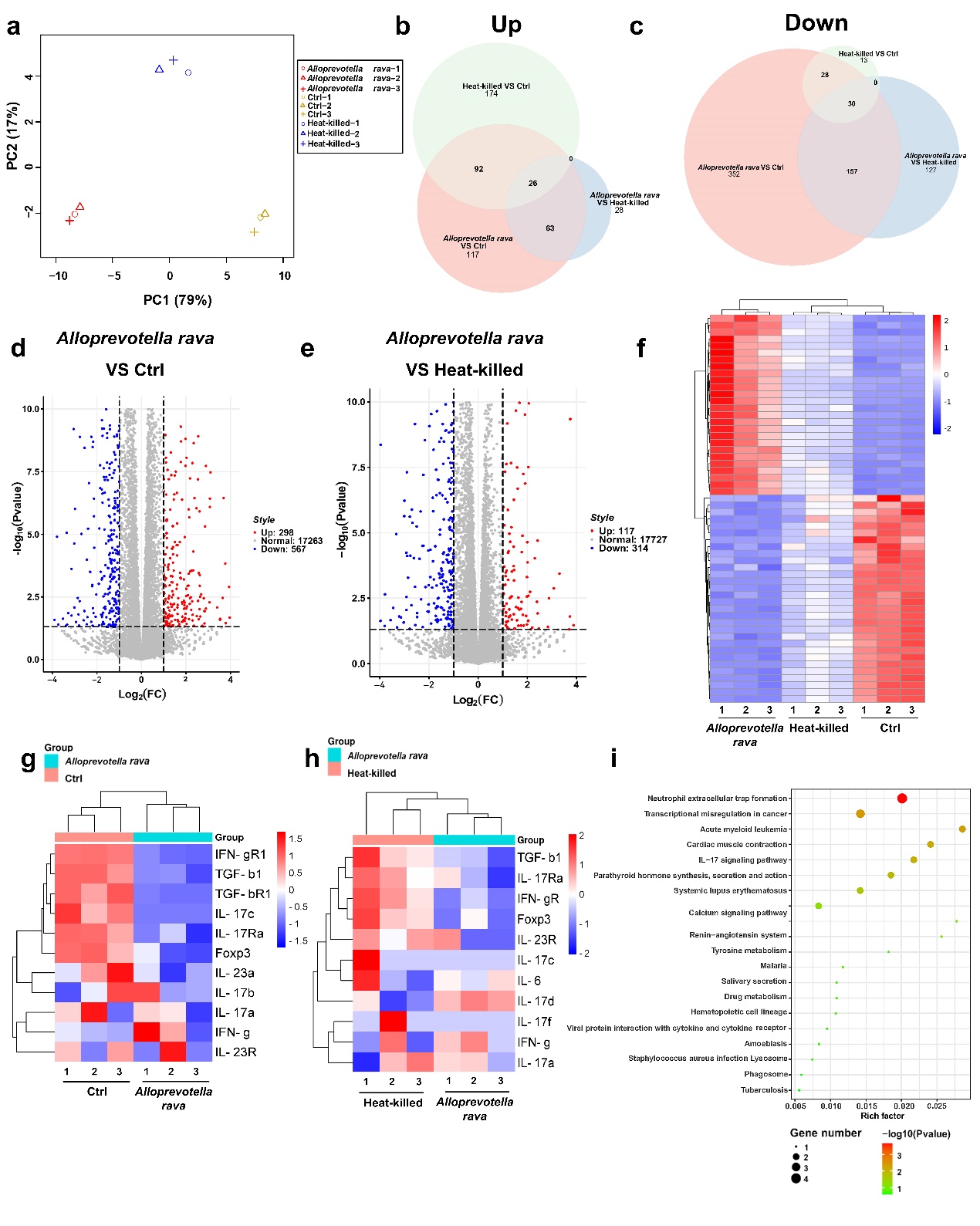


**Figure S6. *Alloprevotella rava* alleviated EAE by inhibiting the Th17 cell differentiation pathway.** (a) PCA analysis between Ctrl, Heat-killed, and *Alloprevotella rava* groups. (b) Venn Analysis of Common Up-regulated Differential Genes Between Ctrl, Heat-killed, and *Alloprevotella rava* groups. (c) Venn Analysis of Common Down-regulated Differential Genes Between Ctrl, Heat-killed, and *Alloprevotella rava* groups. (d) Volcano plot analysis of differentially expressed genes between Ctrl and *Alloprevotella rava* groups. (e) Volcano plot analysis of differentially expressed genes between Heat-killed and *Alloprevotella rava* groups. (f) Heatmap analysis of differentially expressed genes between Ctrl, Heat-killed, and *Alloprevotella rava* groups. (g) Heatmap analysis of Th17 differentiation-related genes between Ctrl and *Alloprevotella rava* groups. (h) Heatmap analysis of Th17 differentiation-related genes between Heat-killed and *Alloprevotella rava* groups. (i) Kyoto Encyclopedia of Genes and Genomes (KEGG) analysis of significantly regulated genes between Ctrl, Heat-killed, and *Alloprevotella rava* groups.


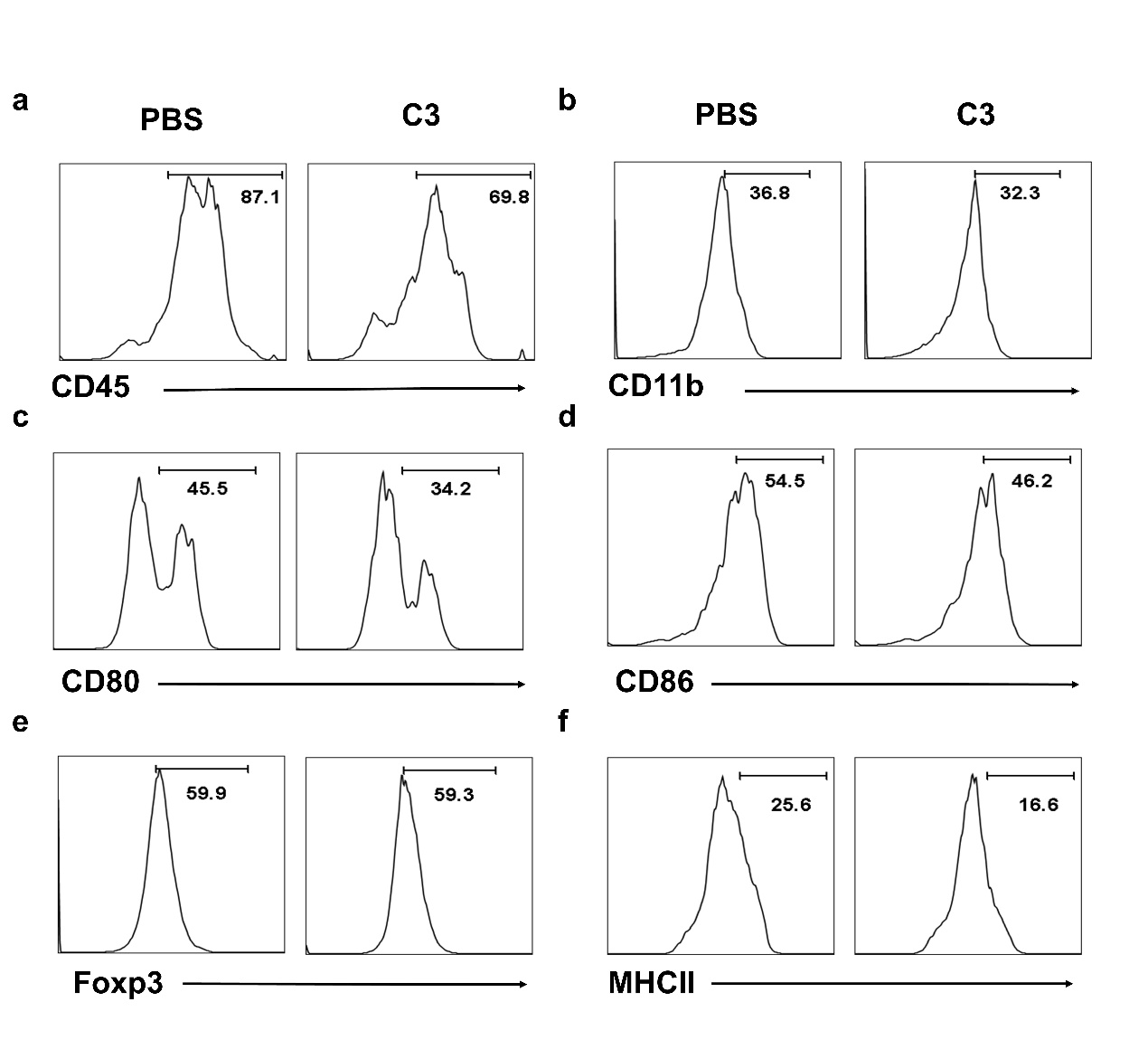


**Figure S7. C3 treatment reduced inflammatory infiltration in the CNS.** Spinal cords and brains were collected from C3- or PBS-treated mice, and MNCs were isolated (*n* = 5 mice in each group). Percentages of (a) CD45^+^, (b) CD11b^+^, (c) CD80^+^, (d) CD86^+^, (e) Foxp3^+^ and (f) MHCII^+^cells were assessed by flow cytometry. Data are expressed as mean ± SEM. One representative of three independent experiments is shown.

**
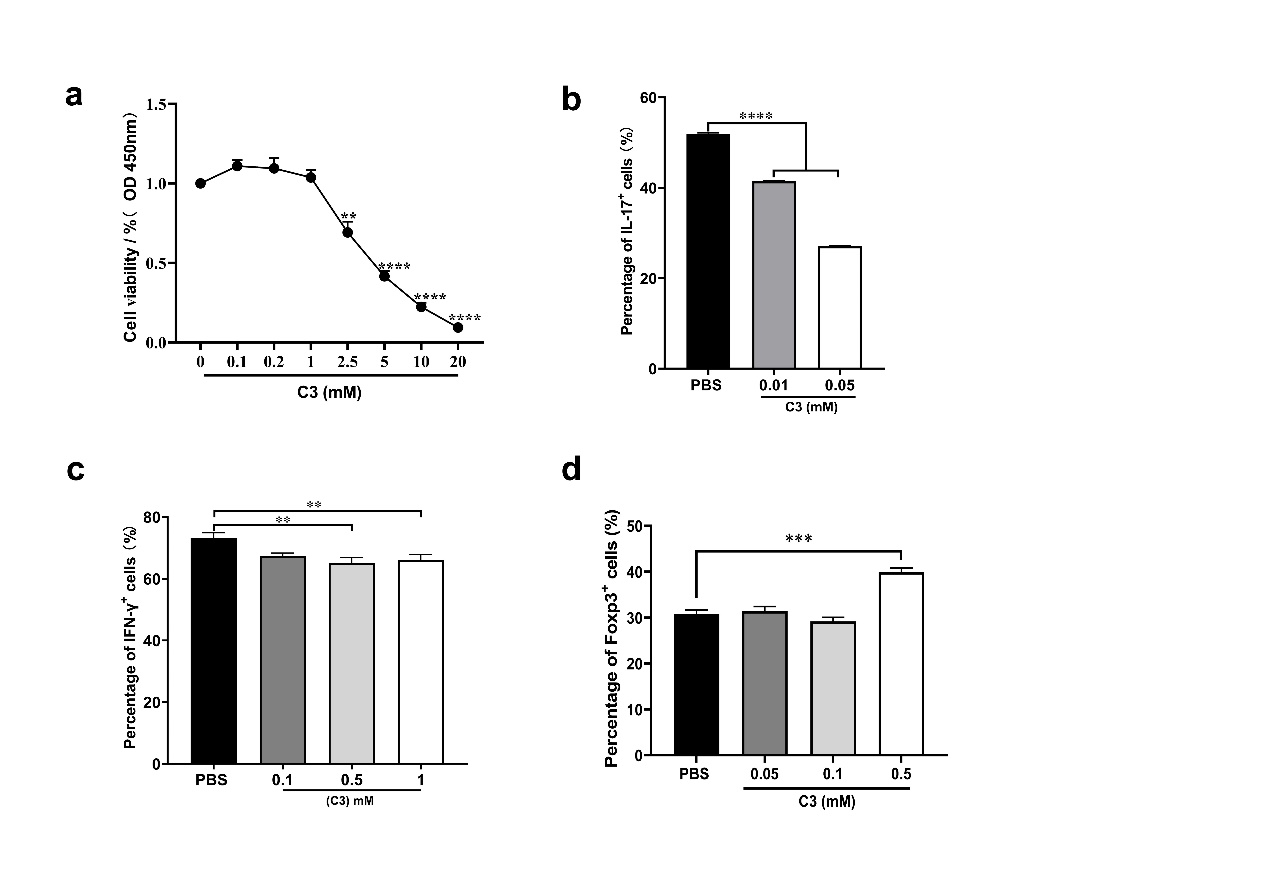
**

**Figure S8. Effects of C3 on Th1, Th17, or Treg differentiation *in vitro*.** (a) Splenocytes were cultured with different concentrations (0-20 mM) of C3 for 18 h. Cell viability was determined by CCK8 assay at 450 nm. (b) CD4^+^ T cells were treated with C3 (0.01 or 0.05 mM) for 72 h under the Th17 polarization condition. IL-17 expression was analyzed by flow cytometry. (c) CD4^+^ T cells were treated with C3 (0.1, 0.5, or 1 mM) for 72 h under the Th1 polarization condition. IFN-γ^+^ expression was analyzed by flow cytometry. (d) CD4^+^ T cells were treated with C3 (0.05, 0.1, or 0.5 mM) for 72 h under the Treg polarization condition. Foxp3^+^ expression was analyzed by flow cytometry. ***p* < 0.01, ****p* < 0.001 and *****p* < 0.0001, Data are expressed as mean ± SEM (*n* = 3 each group), determined by two-way ANOVA (a), or one-way ANOVA (b-d). One representative of three independent experiments is shown.
