# Supplementary material for: Dietary ellagic acid therapy for CNS autoimmunity: Targeting on Alloprevotella rava and propionate metabolism
Source: Microbiome. 2024 Jun 24;12:114. doi: 10.1186/s40168-024-01819-8 (PMC11194905; doi:10.1186/s40168-024-01819-8)
Supplement: Supplementary file 3 — Supplementary Material 2. [file 40168_2024_1819_MOESM2_ESM.docx]

**Supplementary Information for**

**Materials and methods**

***EAE induction, drug dissolution, and treatment***

Female C57BL/6J mice were purchased form the Fourth Military University (Xi’an, China). The protocol and procedures of this experiment were approved by the Institutional Animal Care and Use Committee of Shaanxi Normal University. EAE induction and scoring rules followed previous studies[1]. EA was purchased from (J&K Scientific Ltd,) and dissolved in a specific solvent, and the solvent (Vehicle) consisted of DMSO (3%, Sigma Aldrich), Kolliphor-EL (10%, Sigma Aldrich) and 5% (w/v) Dextrose solution (87%, Sigma Aldrich). C3 was purchased from (Sigma Aldrich) and dissolved in sterile phosphate buffered saline (PBS). EAE mice were randomly divided into the following treatment groups: 1) control group: Vehicle gavage or PBS gavage; 2) EA treatment group: EAE mice were orally administered EA (25 mg/kg/d) at the beginning of immunization (day 0) until the end of the observation period; 3) C3 treatment group: different doses (50, 150, 250 mg/kg/d) of C3 were orally administered at the beginning of immunization (day 0) until the end of the observation period of EAE mice; 4) EAE mice were administered 6.2×10^3^ cfu/mL of *Alloprevotella rava* (BeNa Culture Collection, Strain No. DSM 22548, Cat #: BNCC358370) and heat-killed *Alloprevotella rava* by oral gavage.

***Histological analysis***

EAE mice were sacrificed on 30 d.p.i. and transcardially perfused with autoclaved PBS. Cervical enlargement of spinal cord was harvested for pathological assessment. Cervical enlargements of the spinal cord were cut into 5 μm sections, fixed with 4% paraformaldehyde, and stained with hematoxylin and eosin (H&E) for assessment of inflammation, and with Luxol fast blue (LFB) for demyelination. The sections were assessed and scored in a blinded fashion for inflammation: 0, none; 1, few inflammatory cells; 2, organization of perivascular infiltrates; and 3, abundant perivascular cuffs with extension into adjacent tissue. For demyelination quantification, total white matter was manually delineated and demyelination area (%) was calculated using Image-Pro Plus software.

***Preparation of infiltrating MNCs in CNS***

Spleens were mechanically isolated using a 100 µm cell strainer and incubated with red blood cell lysis buffer for approximately 1 min. Cells were harvested by washing with cold autoclaved PBS before *in vitro* stimulation. CNS cells were obtained using a Nerve dissociation kit (Miltenyi, Cat #: 130-092-628), and spinal cord and brain were mechanically dissociated, passed through a 70 µm cell strainer and washed with cold PBS. Cells were then separated by centrifugation at 2000 rpm for 20 min on a 70/30% Percoll gradient, MNCs were collected from the interface, and washed with PBS.

***Fecal microbiota transplantation and antibiotic treatment***

Feces were collected from Vehicle-treated and EA-treated mice, and fecal samples were resuspended in autoclaved PBS, vortexed and centrifuged for 5 minutes at 4℃. Female C57BL/6 mice aged 6-8 weeks received antibiotic cocktails (vancomycin, 0.5 g/L; neomycin sulfate 1 g/L; metronidazole 1 g/L; ampicillin 1 g/L) dissolved in PBS by gavage once daily for 3 days for gut microbiota disruption [2]. EAE was induced by immunizing 6-8-week-old female recipient mice (n=5 per transplant group) with MOG_35–55_, and FMT was performed daily after immunization. Fresh supernatant (100 μL per mouse) was prepared daily for each recipient mouse by gavage and observed for clinical scores.

Female mice aged 6–8 weeks were randomly divided into four groups: (1) Vehicle group; (2) EA group; (3) antibiotic group; and (4) antibiotic + EA group. EAE was induced by immunization with MOG_35–55_ (n=5 in each group), administered daily after immunization, and observed for clinical scores.

***ELISA***

In a 24-well plate, 1.0 × 10^6^ cells /ml spleen cells were cultured in triplicates in RPMI 1640 containing 10% FBS and cultured for 72 hours with 25 g/mL MOG_35-55_ pulse. The supernatant was collected and tested for IFN-γ, IL-17 and GM-CSF using an ELISA kit (R&D Systems).

C3 content was determined using a Microorganism Propanoic acid (PA) ELISA Kit (Shanghai Win-win Biotechnology Co., Ltd., Cat #: SY-M02935), and after collecting the samples to be tested, the absorbance (OD value) was measured with a microplate reader at a wavelength of 450 nm according to the instructions to calculate C3 concentration.

***FACS analysis***

For surface marker staining, cells were incubated on ice for 30 min with fluorochrome-conjugated antibody or isotype control antibody at the recommended dilution [CD4 (RRID: AB_398528), CD8 (RRID: AB_394081), CD14 (RRID: AB_2740089), CD11b (RRID: AB_ 396636), CD11c (RRID: AB_ 396636), CD80 (RRID: AB_1727514), CD86 (RRID: AB_2075114), MHC II (RRID: AB_394958), CD40 (RRID: AB_2737834), CD206 (RRID: AB_398476), and CD16/32 (RRID: AB_10892816] (BD Biosciences, San Jose, CA). Infiltrating MNCs or splenocytes were activated with 50 ng/mL PMA (Sigma, Cat #: P8139), 500 ng/mL ionomycin (Sigma, Cat #: 13909) for 4h in the presence of 500 ng/mL GolgiPlug (BD Biosciences, Cat #: 512301KZ, RRID: AB_2869014), followed by intracellular staining. Results were parsed using FlowJo software (Treestar, Ashland, OR).

***Western blotting analysis***

Proteins were isolated from cells using PMSF (Invitrogen, Gaithersburg, MD) and protein lysis buffer. The protein concentration was measured by BCA protein assay kit (Solarbio, Cat#: PC0020) and then mixed with SDS sample buffer, boiled and resolved by SDS/PAGE. After electrophoresis, proteins obtained in gels were transferred onto PVDF membranes (Life Technology) and incubated with primary antibodies overnight at 4℃. They were then incubated with HRP-conjugated secondary antibodies and enhanced with Tanon4600 (Shanghai, China) chemiluminescence. Normalization of results was ensured by performing parallel western blot analysis with β-actin antibody (Invitrogen, Cat #: PA1-183, RRID: AB_2539914).

***16S rRNA microbial sequencing analysis***

Fresh fecal samples were collected and stored at -80°C prior to analysis. DNA was extracted from each feces sample using improved protocol based on the Manual for the QIAamp Fast DNA Stool Mini Kit (Qiagen, Germany). The V3-V4 region of the bacterial 16S ribosomal RNA gene was PCR amplified using the barcoded primers, 341F 5’-CCTACGGGRSGCAGCAG-3’ and 806R 5’-GGACTACVVGGGTATCTAATC-3’ (95°C for 3min, followed by 30 cycles of 98°C for 20 s, 58°C for 15s, and 72°C for 20s, with a final extension at 72°C for 5min). PCR reactions were performed in a 30 μL mixture containing 15 μL 2 x KAPA library amplification ReadyMix, 1 μL of each primer (10 μM), 50 ng of template DNA, and ddH_2_O. Amplicons were extracted from 2% agarose gels and the AxyPrep DNA Gel Extraction Kit (Axygen Biosciences, Union City, CA, U.S.) was used according to the manufacturer's instructions. Purification was performed and Qubit®2.0 (Invitrogen, U.S.) was used Quantification was performed. All quantified amplicons were combined and sequenced using Illumina MiSeq/HiSeq (Illumina, Inc., CA, USA) equilibrium concentrations.

Assembled tags, trimmed barcodes and primers, were further checked on their rest lengths and average base quality. 16S tags were restricted between 220 bp and 500 bp such that the average Phred score of bases was no worse than 20 (Q20) and no more than 3 ambiguous N. The copy number of tags was enumerated and redundancy of repeated tags were removed. Only the tags with a frequency of more than 1, which tend to be more reliable, were clustered into OTUs, each of which had a representative tag. Operational Taxonomic Units (OTUs) were clustered with 97% similarity using UPARSE（http://drive5.com/uparse/) and chimeric sequences were identified and removed using Usearch (version 7.0.1090). Each representative tag was assigned to taxa by RDP Classifer (http://rdp.cme.msu.edu/) against the RDP database (http://rdp.cme.msu.edu/) using a confidence threshold of 0.8. OTU profiling table and alpha diversity analyses were also achieved by python scripts of QIIME (version 1.9.1). All the samples were rarified to 474 counts for further statistics. Specific taxa comparisons among groups were analyzed using linear discriminant analysis effect size (LEfSe) and the analysis of the composition of microbiomes (ANCOM), with *p* < 0.05 considered to be a significant difference.

***Fecal sample preparation and targeted metabolomics analysis***

Metabolomics profiling targeting gut microbiota related metabolites were performed. Metabolite identification and quantification were achieved using automated high-throughput metabolite array technology and targeted metabolome batch quantification (TMBQ) software [3]. All samples were stored at -80 ℃ prior to analysis. Firstly, the fecal samples were lyophilized, and about 5 mg of each sample were weighed and transferred into a safety lock tube. Homogenization with 20 μl of ultrapure water was followed by extraction with 120 μL of internal standard solution, followed by centrifugation at 13500 g and 4 ℃ for 10 min. 30 µL of the supernatant were transferred to a 96-well plate for derivatization. Fecal samples were thawed on ice to reduce degradation. 30 μL of the working standard solution and fecal extract were added to a 96-well plate. The following procedures were then performed on an EppendorfepMotion workstation (EppendorfInc., Humburg, Germany). 20 μL of freshly prepared derivatization reagent was added to each well, and after derivatization at 30 ℃ for 60 min, 400 μL of ice-cold 50% methanol solution was added to dilute the sample, followed by 20 min at -20 ℃. This was followed by centrifugation at 4000 g for 30 min at 4 ℃. 135 μL of the supernatant from each well was transferred to a new 96-well plate. Finally, LC-MS analysis was performed. The raw data files generated by UPLC-TQMS were processed using TMBQ software (v1.0, HMI, Shenzhen, Guangdong, China) for peak integration, calibration, and quantification of each metabolite. A total of 132 metabolites were quantified and were subjected to further analysis. Differences between EA *vs.* EAE mice were assessed by using Student’ t test with *p* < 0.05 considered statistically significant.

***Integrative analysis***

For pathological symptoms of EAE mice (*n*=24), the relative abundances of gut microbiota at family and genus level (*n*=65), and gut microbiota related metabolites (*n*=132) were subjected to a random forest algorithm incorporated into a repeated double cross validation framework with unbiased variable selection on each dataset to effectively determine a minimum set of features predicting EA from EAE in a multivariate manner with minimum risk of statistical overfitting [4] (R package ‘MUVR’). All-relevant key features from each dataset were optimally selected. To gain a robust and reliable estimate of model performance, 100 repetitions of the outer cross validations loop was performed, followed by permutation analysis (n=1000) [4, 5]. A multivariate dimension reduction method, DIABLO (Data Integration Analysis for Biomarker discovery using a Latent component method for Omics, R package “mixOmics”), was then employed on key features identified from pathological markers of EAE, gut microbiota and gut microbiota related metabolites. This method has been widely used to identify biologically relevant and highly correlated signatures from various datasets. A random use of full design matrix was applied to look for linear combinations of variables from each omics data set that were maximally correlated. A tuning procedure was applied to determine the optimal number of key variables in each data set to be selected with a minimum misclassification rate. Model performance was then evaluated by a 5-fold cross validation.

***RNA-seq sequencing***

Total RNA was extracted according to the Cell Total RNA Extraction Kit (TIANGEN). mRNA was purified from total RNA using poly-T oligo-attached magnetic beads. Fragmentation was carried out using divalent cations under elevated temperature in NEBNext First Strand Synthesis Reaction Buffer (5×). First strand cDNA was synthesized using random hexamer primer and M-MuLV Reverse Transcriptase (RNase H^-^). Second strand cDNA synthesis was subsequently performed using DNA Polymerase I and RNase H. Remaining overhangs were converted into blunt ends via exonuclease/polymerase activities. After adenylation of 3' ends of DNA fragments, NEBNext Adaptor with hairpin loop structure were ligated to prepare for hybridization. In order to select cDNA fragments of preferentially 250~300 bp in length, the library fragments were purified with AMPure XP system (Beckman Coulter, Beverly, USA). Then 3 µl USER Enzyme (NEB, USA) was used with size-selected, adaptor-ligated cDNA at 37°C for 15 min followed by 5 min at 95 °C before PCR. Then PCR was performed with Phusion High-Fidelity DNA polymerase, Universal PCR primers and Index (X) Primer. At last, PCR products were purified (AMPure XP system) and library quality was assessed on the Agilent Bioanalyzer 2100 system. The clustering of the index-coded samples was performed on a cBot Cluster Generation System using TruSeq PE Cluster Kit v3-cBot-HS (Illumina) according to the manufacturer’s instructions. After cluster generation, the library preparations were sequenced on an Illumina Novaseq6000 platform and 150 bp paired-end reads were generated. The data analysis included normalization of raw data, comparative analysis between samples, gene expression analysis, differential expression gene screening, GO/KEGG pathway enrichment analysis of differentially expressed genes, etc. DESeq2 algorithm was applied to filter differentially expressed genes, and the following criteria were met after significance analysis and FDR analysis: i) | log2FC | > 1; ii) P value < 0.05.

***Determining colonisation of Alloprevotella rava***

We collected feces from mice treated with antibiotics for 3 days, *Alloprevotella rava* gavage for 3 and 7 days. Fecal genomic DNA was extracted with the Fecal Genome Extraction Kit (TIANGEN, Cat #: DP328). Quantitative PCR tested by QuantStudio 3 (Thermo Fisher Scientific, Rockford, MA) and the QuantiFast SYBR Green PCR Kit (Qiagen). Relative expression levels were determined using the ΔΔCt method as previously described. Primers included *Alloprevotella rava* primer (F: GTCGAGGGGAAACGACATTG, R: CTCCCGTAGGAGTTTGGACC) and 16S primer (F: AGAGAGTTTGATCCTGGCTCAG, R: GGTTACCTTGTTACGACTT).

***EA co-cultured with Alloprevotella rava in vitro***

*Alloprevotella rava* (6.2×10^3^ cfu/mL) was co-cultured with sterile water or EA (5 mg/ml) on Columbia blood agar plates (BeNa Culture Collection, Cat #: BNCC352241). Cultures were performed anaerobically at 37 °C.

***CD4^+^ T cells isolation and culture***

Spleens were collected from 6- to 8-week-old C57BL/6 female mice to obtain single-cell mixes. Naïve CD4^+^ T cells were cultured for 72 h with anti-CD3 (0.5 μg/mL) (Bioxcell, Cat #: BE0002, RRID: AB_1107630) and anti-CD28 (1 μg/mL) (Bioxcell, Cat #: BE0015, RRID: AB_1107628). Differentiation into Th17 cells was induced by adding anti-IL-4 (10 μg/mL) （Bioxcell, Cat #: BE0045）, anti-IFN-γ (10 μg/mL) （Bioxcell, Cat #:BE0054）, TGF-β (2 ng/mL)（Peprotech , Cat #: AF-100-21C）, IL-6 (20 ng/mL)（Peprotech , Cat #: #21616-50 UG）, and IL-1β (R&D Systems, 10 ng/mL) (Peprotech, Cat #:201-LB). Differentiation into Th1 cells was induced by adding anti-IL-4 (10 μg/mL) and IL-12 (10 ng/mL) (Peprotech, Cat #:210-12). Differentiation into Treg cells was induced by adding TGF-β (2 ng/mL)（Peprotech , Cat #: AF-100-21C）and IL-1β (10 ng/mL) (R&D Systems, Cat #:201-LB).

***HDAC activity assay***

CD4^+^ T cells were treated with anti-CD3 (Bioxcell, Cat #: BE0002, RRID: AB_1107630) and anti-CD28 mAb (Bioxcell, Cat #: BE0015, RRID: AB_1107628) for 24h under Th17 conditions in the presence or absence of C3 (1 mM) or TSA (0.5 μM) (MedChemExpress, Cat #: HY-15144). Cells were collected. Nuclear proteins were extracted using Nuclear Protein Extraction Kit (Solarbio, Cat #: R0050). Then, the nuclear protein samples were incubated with HDAC Green substrate (AAT Bioquest, Cat #: 13601) at 37°C for 40 min, and then the fluorescence intensity at excitation/emission (490/525 nm) was measured.

***Statistical analysis***

Other than gut microbiota and metabolites data, other data are expressed as mean ± SEM. Statistical differences between two or more groups were achieved using unpaired Student’s t-test or ANOVA using GraphPad Prism 8 (GraphPad, La Jolla, CA). It was considered that the difference with P value less than 0.05 was statistically significant.

**References**

1. Zhang Y, Li X, Ciric B, Curtis MT, Chen WJ, Rostami A, et al. A dual effect of ursolic acid to the treatment of multiple sclerosis through both immunomodulation and direct remyelination. Proceedings of the National Academy of Sciences of the United States of America. 2020;117(16):9082-9093.

2. Zeng SL, Li SZ, Xiao PT, Cai YY, Chu C, Chen BZ, et al. Citrus polymethoxyflavones attenuate metabolic syndrome by regulating gut microbiome and amino acid metabolism. Science advances. 2020;6(1):eaax6208.

3. Xie G, Wang L, Chen T, Zhou K, Zhang Z, Li J, et al. A Metabolite Array Technology for Precision Medicine. Analytical chemistry. 2021;93(14):5709-5717.

4. Shi L, Westerhuis JA, Rosén J, Landberg R, Brunius C. Variable selection and validation in multivariate modelling. Bioinformatics. 2019;35(6):972-980.

5. Liu Z, Dai X, Zhang H, Shi R, Hui Y, Jin X, et al. Gut microbiota mediates intermittent-fasting alleviation of diabetes-induced cognitive impairment. Nature communications. 2020;11(1):855.
