# Supplementary material for: Dietary ellagic acid therapy for CNS autoimmunity: Targeting on Alloprevotella rava and propionate metabolism
Source: Microbiome. 2024 Jun 24;12:114. doi: 10.1186/s40168-024-01819-8 (PMC11194905; doi:10.1186/s40168-024-01819-8)
Supplement: Supplementary file 4 — Supplementary Material 3. [file 40168_2024_1819_MOESM3_ESM.docx]

**Supplementary Information for**

**Supplementary table 1**

**Supplementary table 1 Biological activity of key metabolites identified by integrative modelling and their relevant bacteria**

| Metabolite | Associated gut microbiota | | Biological activity |
| --- | --- | --- | --- |
| Heptadecanoic acid [1] | | Prevotella, Lactobacillus, and Alistipes | Activation of GPR40 signaling pathway Promotion of colonic motility in rats |
| Suberic acid [2, 3] | | *Acinetobacter* sp. strain SE19, | Reduction of skin photoaging |
| Palmitic acid [4, 5] | | Bacteroidetes | Inhibition of tumor growth, metabolic disturbances and inflammation |
| DPA [6, 7] | | *Dialister*, *Prevotella*, *Finegoldia*, *Anaerococcus*,  *Peptoniphilus*, WAL 1855D,  *Porphyromonas*, *Campylobacter* | Inhibition of inflammation  Favour of obesity, type 2 diabetes, and several cardiovascular diseases |
| *p*-Hydroxyphenylacetic acid [8, 9] | | *Lactobacillus strains*, *Clostridium difficile* | Production of *p*-cresol is bacteriostatic |
| Isovaleric acid [10] | | *Faecalibacterium*, *Alistipes*, *Ruminococcus*, *Ocillibacter*, *Bacteroides dorei*, *Faecalibacterium*, *Ruminococcus*, *Bacteroides uniformis* | Strong relevance of with depression |
| Acetic acid [11-14] | | *Clostridium* clusters IV and XIVa, *Akkermansia muciniphila*,  *Bacteroides*, *Bifidobacterium*, *Prevotella,*  *Ruminococcus* | Improvement of glucose tolerance  Suppression of allergic airway disease |
| Propionic acid [12, 15-20] | | *Bacteroides*, *Phascolarctobacterium succinatutens*,  *Dialister*, *Veillonella*, *Megasphaera elsdenii*, *Coprococcus catus*, *Salmonella*, *Roseburia inulinivorans*, *Ruminococcus obeum*, *Alloprevotella*, *Prevotella* | Improvement of glucose homeostasis  Protection of allergic airways  Increase of secretory activity in the colon  Reduction of inflammation  Reduction of cardiovascular risk |

**References**

1. Zhao L, Huang Y, Lu L, Yang W, Huang T, Lin Z, et al. Saturated long-chain fatty acid-producing bacteria contribute to enhanced colonic motility in rats. Microbiome. 2018;6(1):107.

2. Clomburg JM, Blankschien MD, Vick JE, Chou A, Kim S, Gonzalez R. Integrated engineering of β-oxidation reversal and ω-oxidation pathways for the synthesis of medium chain ω-functionalized carboxylic acids. Metabolic engineering. 2015;28:202-12.

3. Kang W, Choi D, Park T. Dietary Suberic Acid Protects Against UVB-Induced Skin Photoaging in Hairless Mice. Nutrients. 2019;11(12).

4. Hosomi K, Kiyono H, Kunisawa J. Fatty acid metabolism in the host and commensal bacteria for the control of intestinal immune responses and diseases. Gut microbes. 2020;11(3):276-84.

5. Carta G, Murru E, Banni S, Manca C. Palmitic Acid: Physiological Role, Metabolism and Nutritional Implications. Frontiers in physiology. 2017;8:902.

6. García-Mantrana I, Selma-Royo M, González S, Parra-Llorca A, Martínez-Costa C, Collado MC. Distinct maternal microbiota clusters are associated with diet during pregnancy: impact on neonatal microbiota and infant growth during the first 18 months of life. Gut microbes. 2020;11(4):962-78.

7. Tortosa-Caparrós E, Navas-Carrillo D, Marín F, Orenes-Piñero E. Anti-inflammatory effects of omega 3 and omega 6 polyunsaturated fatty acids in cardiovascular disease and metabolic syndrome. Critical reviews in food science and nutrition. 2017;57(16):3421-9.

8. Yokoyama MT, Carlson JR. Production of Skatole and para-Cresol by a Rumen Lactobacillus sp. Applied and environmental microbiology. 1981;41(1):71-6.

9. Selmer T, Andrei PI. p-Hydroxyphenylacetate decarboxylase from Clostridium difficile. A novel glycyl radical enzyme catalysing the formation of p-cresol. European journal of biochemistry. 2001;268(5):1363-72.

10. Szczesniak O, Hestad KA, Hanssen JF, Rudi K. Isovaleric acid in stool correlates with human depression. Nutritional neuroscience. 2016;19(7):279-83.

11. Sivaprakasam S, Prasad PD, Singh N. Benefits of short-chain fatty acids and their receptors in inflammation and carcinogenesis. Pharmacology & therapeutics. 2016;164:144-51.

12. Koh A, De Vadder F, Kovatcheva-Datchary P, Bäckhed F. From Dietary Fiber to Host Physiology: Short-Chain Fatty Acids as Key Bacterial Metabolites. Cell. 2016;165(6):1332-45.

13. Yamashita H, Fujisawa K, Ito E, Idei S, Kawaguchi N, Kimoto M, et al. Improvement of obesity and glucose tolerance by acetate in Type 2 diabetic Otsuka Long-Evans Tokushima Fatty (OLETF) rats. Bioscience, biotechnology, and biochemistry. 2007;71(5):1236-43.

14. Thorburn AN, McKenzie CI, Shen S, Stanley D, Macia L, Mason LJ, et al. Evidence that asthma is a developmental origin disease influenced by maternal diet and bacterial metabolites. Nature communications. 2015;6:7320.

15. De Vadder F, Plessier F, Gautier-Stein A, Mithieux G. Vasoactive intestinal peptide is a local mediator in a gut-brain neural axis activating intestinal gluconeogenesis. Neurogastroenterology and motility : the official journal of the European Gastrointestinal Motility Society. 2015;27(3):443-8.

16. Trompette A, Gollwitzer ES, Yadava K, Sichelstiel AK, Sprenger N, Ngom-Bru C, et al. Gut microbiota metabolism of dietary fiber influences allergic airway disease and hematopoiesis. Nature medicine. 2014;20(2):159-66.

17. Yajima T, Inoue R, Matsumoto M, Yajima M. Non-neuronal release of ACh plays a key role in secretory response to luminal propionate in rat colon. The Journal of physiology. 2011;589(Pt 4):953-62.

18. Kong C, Gao R, Yan X, Huang L, Qin H. Probiotics improve gut microbiota dysbiosis in obese mice fed a high-fat or high-sucrose diet. Nutrition (Burbank, Los Angeles County, Calif). 2019;60:175-84.

19. Downes J, Dewhirst FE, Tanner ACR, Wade WG. Description of Alloprevotella rava gen. nov., sp. nov., isolated from the human oral cavity, and reclassification of Prevotella tannerae Moore et al. 1994 as Alloprevotella tannerae gen. nov., comb. nov. International journal of systematic and evolutionary microbiology. 2013;63(Pt 4):1214-8.

20. Bartolomaeus H, Balogh A, Yakoub M, Homann S, Markó L, Höges S, et al. Short-Chain Fatty Acid Propionate Protects From Hypertensive Cardiovascular Damage. Circulation. 2019;139(11):1407-21.
